# Resolution of the *Portunusgladiator* species complex: taxonomic status and identity of *Monomiagladiator* (Fabricius, 1798) and *Monomiahaanii* (Stimpson, 1858) (Brachyura, Decapoda, Portunidae)

**DOI:** 10.3897/zookeys.858.33826

**Published:** 2019-07-01

**Authors:** Amanda M. Windsor, Jose Christopher E. Mendoza, Jonathan R. Deeds

**Affiliations:** 1 United States Food and Drug Administration, Office of Regulatory Science, 5001 Campus Dr. College Park, MD 20740 United States Food and Drug Administration College Park United States of America; 2 Lee Kong Chian Natural History Museum, Faculty of Science, National University of Singapore, 2 Conservatory Drive, 117377 Singapore National University of Singapore Singapore Singapore

**Keywords:** Commercial species, DNA barcoding, molecular phylogenetics, morphology, seafood, swimming crab, taxonomy

## Abstract

The United States Food and Drug Administration (FDA) has recently adopted DNA barcoding for the purpose of determining the species identity of commercial seafood products. This effort has revealed instances of incongruence between current scientifically accepted taxon names and those utilized by the seafood industry in product labelling. One such case is that of “*Portunushaanii*”, a name utilized by the seafood industry to label commercial products under the market name “red swimming crab.” However, carcinologists currently regard *P.haanii* as synonym of *Portunusgladiator* Fabricius, 1798, which itself is the subject of debate over whether it is a secondary homonym of *Cancer gladiator* Fabricius, 1793. Further complicating matters, DNA barcode sequences from commercial products match GenBank sequences identified as *Portunuspseudoargentatus* Stephenson, 1961. Here the complicated taxonomic history of the *Portunusgladiator* complex is reviewed and a resolution proposed based on combined morphological descriptions and molecular phylogenetic analyses. It is demonstrated that, given the provisions of the International Code of Zoological Nomenclature and the current elevation of *Monomia* Gistel, 1848, to full genus rank, its type species, *Portunusgladiator* Fabricius, 1798, should be treated as a valid and available taxon name. It is also shown, upon examination and comparison of types and topotypic material that *Monomiahaanii* (Stimpson, 1858) is a distinct taxon from *M.gladiator*, and *Portunuspseudoargentatus* Stephenson, 1961, is a junior subjective synonym of *M.haanii* (Stimpson, 1858). Furthermore, it is shown that crab meat sold in the US currently labeled as “*Portunushaanii*” and/or “red swimming crab” is in fact *M.haanii* using comparative analysis of DNA barcode sequences between museum-vouchered reference specimens, whole crabs provided directly by a seafood importer, and processed commercial products purchased at retail.

## Introduction

The United States Food and Drug Administration (FDA) has adopted DNA barcoding for the purpose of species identification to assure the accurate labelling of seafood products as well as to address issues with species substitution and fraud (Handy et al. 2011; Eischeid et al. 2016). This effort has identified instances of incongruence between currently accepted taxon names and the names utilized by industry in product labelling. At the same time that FDA was beginning to build its reference library of decapod crustacean DNA standards (Food and Drug Administration 2017), species substitution of brachyuran crabs was highlighted by a survey of crab cakes from restaurants in the Maryland / Washington, DC metropolitan area (Warner et al. 2015). DNA testing from that survey of products advertised by restaurants to contain crabmeat only from “local” *Callinectessapidus* Rathbun, 1896, revealed at least one of six species of substituted portunoid crab in 38% of the crab cakes tested (Warner et al. 2015). According to the report, the most commonly detected substitute species was *Portunuspelagicus* (Linnaeus, 1758) followed by *P.pseudoargentatus* Stephenson, 1961, and *P.sanguinolentus* (Herbst, 1783). The reported DNA match to *P.pseudoargentatus* was of particular interest to Warner et al. (2015) who noted that this species was, at the time, not included in the FDA Guide to Acceptable Market Names for Seafood Sold in Interstate Commerce (The Seafood List) (Food and Drug Administration 2012) nor in the FAO fishery species list (Food and Agriculture Organization of the United Nations 2010–2018), raising concern that this species was unknown to regulators. As a result, in addition to the existing entry for *P.haanii*, *P.pseudoargentatus* was added to The Seafood List in 2015 (Food and Drug Administration 2015). Concerns over an unknown species that was apparently common in the US food supply led us to further investigate the identity of *P.pseudoargentatus* and its close relatives within the *Portunusgladiator* species complex to determine their relationship to what was currently being harvested and marketed as “*P.haanii*” and/or “red swimming crab.”

At the time of the study by Warner et al. (2015), only three sequences from three loci belonging to one specimen identified as “*Portunuspseudoargentatus* (ZMMU Ma 3368),” which was collected from Nhatrang Bay, Vietnam, had been deposited in GenBank (JX398121, JX398079, JX398094) (Spiridonov et al. 2014). Further complicating matters, there is a set of DNA barcode sequences (BOLD: AAO6694) identified as “*Portunushaanii*” from the Coral Sea in the Barcode of Life Database (BOLD), which led to more uncertainty about the true identity of crabs in the US seafood supply. The specimens in question, which are deposited in the Muséum national d’Histoire naturelle (MNHN) in Paris under the revised name of *P.gladiator* (MNHN-IU-2008-12570-77), have since been examined by one of the present authors (AMW) and confirmed to be neither *P.gladiator* nor *P.pseudoargentatus*, but morphologically and genetically closer to *Monomialucida* Koch and Ďuriš, 2017. *Portunushaanii* was included on the FDA Seafood List prior to this study without knowledge that *P.haanii* is currently an unaccepted species name, having been synonymized under *P.gladiator* Fabricius, 1798 (Stephenson and Cook 1973, Ng et al. 2008).

Within the seafood industry, *Portunushaanii* and/or “red swimming crab” is the species/market name used for crabs harvested extensively from China and Vietnam (Monterey Bay Aquarium Seafood Watch 2013; Fishsource 2016). Photographs identified as *P.haanii* on websites advertising “*Portunushaanii*” (e.g., Fishsource 2016; Alibaba.com 2018) bear a striking resemblance to the color photograph of “*Portunuspseudoargentatus* (ZMMU Ma 3368)” (Chertoprud et al. 2012: pl. 51 fig. H) and Stephenson’s (1961) original description of *P.pseudoargentatus*. The names *Portunusgladiator* and *Portunuspseudoargentatus* were not found associated with any specific crabmeat products in neither our on-line searches nor our discussions with industry representatives.

### Taxonomic history

The taxonomy of the *Portunusgladiator* complex is so convoluted that it makes a chronologically arranged taxonomic history difficult to compile. Here, we present the significant taxonomic actions for *P.gladiator*, *P.haanii*, and *P.pseudoargentatus*.

Fabricius (1798) described a swimming crab collected from “Oceano Asiatico Dom. Daldorff” (probably Tranquebar, India; see Ng et al. 2008), which he named *Portunusgladiator*, based on an unspecified quantity of specimens. Fabricius (1798: 368) gave a brief description in Latin, stating: “P. thorace tomentoso utrinque novemdentato: dente postico maiore, minibus sanguineo maculatis. Habitat in Oceano Asiatico Dom. Daldorff. Praecedentibus affinis at minor. Thorax holosericeus, parum inaequalis, hine inde scaber. Chelae sanguineo maculatae digitis apice dentibusque albis.”

Previously, however, Fabricius (1793) had given the same specific epithet to a different species of swimming crab (from “Nova Hollandia” = Australia), *Cancer gladiator*. Fabricius (1793: 449) provided this description: “C. thorace laevi: lateribus octodentatis, postico maximo, minibus angulatis. Cancer hastatus. Mant. Ins. r. 319. 34. Habitat in Nova Hollandia Mus. Dom. Banks. Minutus pullo modo Cancer hastatus Linnaei. Palmae anticae bidentatae, chelae angulatae. Palmae posticae angulatae.”

Latreille (1825) in his treatment of *Portunus*, considered *Cancer gladiator* Fabricius, 1793, a junior synonym of *Portunussanguinolentus* (Herbst, 1783), and clearly also considered *Portunusgladiator* Fabricius, 1798, to be a distinct species. From this point on, it appears that the name “*Portunusgladiator*” has been almost exclusively associated with the species described in 1798. Stephenson and Cook (1973) would later accept Latreille’s (1825) synonymization and selected a male specimen of *P.sanguinolentus* from Queensland, Australia (QM W3683) to be the neotype of *C.gladiator* Fabricius, 1793, in order to stabilize its taxonomy.

De Haan (1833) then established the subgenus Portunus (Amphitrite), to which Miers (1886) subsequently designated Neptunus (Amphitrite) gladiator (Fabricius, 1798), as its type species. Prior to Miers’ action, however, Gistel (1848) had proposed a replacement name, Portunus (Monomia), for this subgenus as the name *Amphitrite* had been previously used for a polychaete annelid genus, viz. *Amphitrite* Müller, 1771. By virtue of these, *Portunusgladiator* Fabricius, 1798, is the type species of Portunus (Monomia) Gistel, 1848.

Stimpson (1858) gave the name *Amphitritehaanii* to a species from Tanegashima and Kagoshima, Japan and the ‘China Seas, above 23°N latitude,’ previously identified by De Haan (1833, 1835) as Portunus (Amphitrite) gladiator (Fabricius, 1798). Stimpson (1858: 38) wrote: “Amphitrite Haanii. *A.gladiator*, De Haan; loc. cit. p. 29, pl. i. f. 5. (v ix *L.gladiator*, M. Edwards.) In mari Sinensi, lat. bor. 23°; ad insulam ‘Tanegasima’; et in sinu ‘Kagosima’; in fundis arenosis prof. 12-20 org.”

As Stimpson (1858) had material from Japan and also referred to De Haan’s (1833) citations of the species as P. (A.) gladiator, all of Stimpson’s and De Haan’s specimens are effectively syntypes. Later, Stimpson (1907: 79) provided a slightly more detailed explanation, clearly opining that De Haan’s (1833, 1835) Japanese *P.gladiator* was a distinct species from H. Milne Edwards’ (1834)*Lupeagladiator* (= *P.gladiator* Fabricius, 1798) from the Indian Ocean. There are 53 extant syntypes of *Amphitritehaanii* in the collection of the Naturalis Biodiversity Center in Leiden as recorded by Yamaguchi and Baba (1993), and among these they selected a male specimen (RMNH 379, CW = 42 mm, CL = 24 mm, C. Fransen pers. comm.) to be the lectotype.

Stephenson (1961) described *Portunuspseudoargentatus* based on one male specimen from the Abrolhos Islands, off the western coast of Australia. He cited differences in the morphology of the male 6^th^ pleomere and male gonopod 1 (G1) between *P.gladiator* and the new species. Crosnier (1962) in his treatment of the swimming crabs of Madagascar commented on the confusion in the identities of specimens from various localities labelled as “*P.gladiator*.” He highlighted the differences in the morphology of the male 6^th^ pleomere and G1, wherein *P.pseudoargentatus* tends to have the male 6^th^ pleomere with less sinuous lateral borders, and the G1 more greatly bent in the middle (midlength) compared to *P.gladiator*. He also referred to Japanese material that he examined to *P.pseudoargentatus*, apparently not considering Stimpson’s (1858, 1907) earlier reports on *P.haanii*.

Stephenson and Cook (1973) did an extensive study on the *Portunusgladiator* complex, and they argued that, with both being then classified in the genus *Portunus*, *P.gladiator* Fabricius, 1798, effectively became a secondary homonym of *P.gladiator* (Fabricius, 1793) (= *P.sanguinolentus*), and a replacement name was needed. They went on to suggest *Amphitritehaanii* Stimpson, 1858, as the earliest available replacement name, eliminating an earlier name, *Cancer menestho* Herbst, 1803, on the basis of a difference in colouration and in the armature of the cheliped merus in the illustration of the latter (viz. Herbst, 1803:pl. 55 fig. 3). They also considered *Portunuspseudoargentatus* Stephenson, 1961, as a junior synonym of *Portunushaanii* (Stimpson, 1858). Additionally, Stephenson and Cook (1973) described aberrant “forms” within *P.haanii*, on the basis of the G1 morphology (e.g., “normal” vs. “unusual” specimens; viz. Stephenson and Cook 1973: figs 6, 7). These “unusual” specimens were two crabs from the Bay of Jeddo, Japan, and the holotype of *P.pseudoargentatus*. They did not, however, take steps to formally distinguish these forms as separate species, subscribing instead to the concept of “*Portunushaanii*” as a morphologically variable species.

Ng et al. (2008: 156, 157) provided a detailed account on the nomenclature of Fabricius’ two species with the epithet *gladiator*, essentially stating that, contrary to Stephenson and Cook (1973), there is no secondary homonymy unless *Cancer gladiator* is considered a distinct species from *Portunussanguinolentus*: “… as *Cancer gladiator* Fabricius, 1793, is regarded as a junior synonym of *Portunussanguinolentus* (Herbst, 1783), the name ‘*Portunusgladiator* (Fabricius, 1793)’ has not been recognized or used anywhere. This being the case, there is no homonymy with *Portunusgladiator* Fabricius, 1798, and this name should remain available for use under the Code. The issue of secondary homonymy will only arise if *Cancer gladiator* Fabricius, 1793, is regarded as a valid species of *Portunus* distinct from *Portunussanguinolentus* (Herbst, 1783). If this were to happen (for example, if the widespread *P.sanguinolentus* was to prove to be a complex of several cryptic species), then the name *Portunusgladiator* Fabricius, 1798, would have to be replaced by the next available name…”. Ng et al. (2008), however, maintained the synonymy of *Portunusgladiator* and *Amphitritehaanii*, while still considering *Portunuspseudoargentatus* a valid species.

Chertoprud et al. (2012) in their report on the commercially valuable brachyuran species of Vietnam, commented that Stephenson and Cook’s (1973) mention of “*Portunusgladiator* (Fabricius, 1793)” was sufficient to activate Article 59 of the Code (ICZN 1999), which therefore necessitated the use of *Portunushaanii* (Stimpson, 1858) as a replacement name for *Portunusgladiator* Fabricius, 1798.

As things stand, the issue on the validity of the names “*Portunusgladiator*” and “*Portunushaanii*” has not been satisfactorily settled. Recent publications on the systematics of Portunidae have bolstered the concept of *Monomia* Gistel, 1848, as a valid genus-level taxon distinct from *Portunus*, but these have also shown that the problem with the taxonomy of the type species, *M.gladiator* (Fabricius, 1798), and its closely related congeners, *M.haanii* (Stimpson, 1859) and *M.pseudoargentata* (Stephenson, 1961), remains unresolved (Chertoprud et al. 2012; Spiridonov et al. 2014; Koch et al. 2017; Koch and Ďuriš 2018). This problem, unless addressed, is expected to have wide-ranging consequences on the taxonomy of *Monomia*. As such, the identities of *M.gladiator*, *M.haanii*, and *M.pseudoargentata* need to be firmly established.

An integrative approach with morphological and molecular phylogenetic analyses was undertaken to resolve and stabilise the taxonomy of the *Portunusgladiator* (=*Monomiagladiator*) complex. The molecular results of the morphologically verified and vouchered reference specimens, which included whole specimens from Asian fish ports and a seafood importer, were then used as standards to identify the contents of cans of pasteurized lump crabmeat labeled as “*Portunushaanii*” and/or “red swimming crab” through comparative analysis of DNA barcode sequences.

## Materials and methods

### Taxonomic methods

Materials examined are deposited at the US National Museum of Natural History, Smithsonian Institution (USNM); Florida Museum of Natural History, University of Florida (UF); Western Australian Museum (WAM); and Lee Kong Chian Natural History Museum, National University of Singapore (ZRC). These included the holotype of *Portunuspseudoargentatus* (WAM-C7506), as well as crabs purchased at fish ports in India, Thailand, and Taiwan, and whole crabs identified by a US seafood importer as “*Portunushaanii*.” Photographs of the type specimens of *Portunusgladiator* and *Amphitritehaanii*, housed at Zoological Museum at the University of Copenhagen (ZMUC) and Naturalis Biodiversity Center, Leiden (RMNH), respectively, were also examined. Details on all specimens utilized in morphological examinations are provided in the material examined subsection of the taxonomic account below. The morphological terminology largely follows Wee and Ng (1995) and Apel and Spiridonov (1998). The following abbreviations are used:

**CL** carapace length, taken along the dorsal midline from the tips of the frontal teeth to the posterior margin of the carapace;

**CW** carapace maximum width, taken at the level of its widest point;

**P1–P5** first to fifth pereopods, respectively (P1, chelipeds; P2–P5, first to fourth ambulatory legs);

**G1, G2** first and second male pleopods, respectively.

The term, pleomere (first to sixth), here refers to the six somites of the pleon. When possible, DNA was extracted from the specimens utilized for the morphological studies. Details on all specimens utilized in the molecular phylogenetic component of this study are given in Table 1, Nomenclatural decisions are based on the provisions of the International Code of Zoological Nomenclature, here referred to as “the Code” (ICZN 1999).

**Table 1.** Material examined in molecular analyses with details on voucher identification numbers, sex, country in which the specimen was collected, the fish port or body of water, and pertinent GenBank Accession Numbers. Voucher ID abbreviations: IOM= Institute of Oceanology and Museum, Nha Trang; MNHN= Muséum National d’Histoire Naturelle, Paris; NHMUK= The Natural History Museum, London; UF= University of Florida Natural History Museum, Gainesville; UO= University of Ostrava, Ostrava; USNM= United States National Museum, National Museum of Natural History, Washington, D.C.; WAM= Western Australian Museum, Perth; ZMMU= Zoological Museum of the Moscow University, Moscow; ZRC= Zoological Reference Collection, Lee Kong Chian Natural History Museum, Singapore.

Taxon name	Voucher ID	Sex	Country	Port/Body of Water	GenBank Accession Numbers		
					12S	16S	COI
*Monomiagladiator*	ZRC 2016.0145	M	India	Pazhayar Fish Landing, Bay of Bengal	MK270964	—	MK281257
*Monomiagladiator*	ZRC 2016.0149	F	India	Pondicherry, Bay of Bengal	MK270959	MK271060	MK281259
*Monomiagladiator*	WAM C61156	F	Australia	Pilbara Shelf, Indian Ocean	MK270957	MK271053	MK281253
*Monomiagladiator*	WAM C26459	M	Australia	Dampier Archipelago, Cape Brugieres	MK270956	MK271047	MK281247
*Monomiagladiator*	UF 36251	F	Singapore	Singapore Strait	MK270963	MK271029	MK281229
*Monomiagladiator*	USNM 127068	F	Thailand	Andaman Sea	MK270962	MK271030	MK281230
*Monomiagladiator*	ZRC 2000.0842	M	Thailand	Pichai Fish Port, Phuket, Andaman Sea	MK270958	—	MK281230
*Monomiagladiator*	ZRC 2003.0114	M	Thailand	Pattani Fish Port, Gulf of Thailand	MK270960	MK271055	
*Monomiagladiator*	ZRC 2003.0197	M	Thailand	Saiburi Crab Landing, Gulf of Thailand	MK270961	MK271056	
*Monomiagladiator*	ZRC 2002.0297	F	Thailand	Pichai Fish Port, Phuket, Andaman Sea	—	—	
*Monomiagladiator*	MNHN-IU-2014-10087		Vietnam	Của Bé Fishing Port	—	KY524466 ^1^	—
*Monomiagladiator*	ZMMU Ma 3366		Vietnam	Của Bé Fishing Port	—	—	JX398095 ^2^
*Monomiahaanii*	ZRC 1999.0084	M	Japan	Pacific Ocean	MK270948	MK271054	MK281255
*Monomiahaanii*	WAM C61155	F	Australia	Pilbara Shelf	MK270946	MK271052	MK281252
*Monomiahaanii*	WAM C34767	F	Australia	Exmouth Gulf	MK270944	MK271048	MK281248
*Monomiahaanii*	WAM C34900	M	Australia	Exmouth Gulf	MK270945	MK271049	MK281249
*Monomiahaanii*	WAM C44737	F	Australia	Ningaloo Marine Park	MK270954	MK271051	MK281251
*Monomiahaanii*	WAM C34938	F	Australia	Shark Bay	MK270949	MK271050	MK281250
*Monomiahaanii*	WAM C55510	M	Australia	Shark Bay	MK270943	MK271045	MK281245
*Monomiahaanii*	WAM C7506*	M	Australia	Abrolhos Islands	MK270953	MK271046	MK281246
*Monomiahaanii*	USNM 1421161	M	China	South China Sea, FAO Fishing Area 61	MK270934	MK271033	MK281233
*Monomiahaanii*	USNM 1421181	M	China	South China Sea, FAO Fishing Area 61	MK270935	MK271034	MK281234
*Monomiahaanii*	USNM 1421182	M	China	South China Sea, FAO Fishing Area 61	MK270936	MK271035	MK281235
*Monomiahaanii*	USNM 1421185	M	China	South China Sea, FAO Fishing Area 61	MK270937	MK271036	MK281236
*Monomiahaanii*	USNM 1421187	M	China	South China Sea, FAO Fishing Area 61	MK270950	MK271037	MK281237
*Monomiahaanii*	USNM 1421191	M	China	South China Sea, FAO Fishing Area 61	MK270938	MK271038	MK281238
*Monomiahaanii*	USNM 1421194	M	China	South China Sea, FAO Fishing Area 61	MK270951	MK271039	MK281239
*Monomiahaanii*	USNM 1421195	M	China	South China Sea, FAO Fishing Area 61	MK270939	MK271040	MK281240
*Monomiahaanii*	USNM 1421196	M	China	South China Sea, FAO Fishing Area 61	MK270940	MK271041	MK281241
*Monomiahaanii*	USNM 1421202	M	China	South China Sea, FAO Fishing Area 61	MK270941	MK271042	MK281242
*Monomiahaanii*	USNM 1421204	M	China	South China Sea, FAO Fishing Area 61	MK270952	MK271043	MK281243
*Monomiahaanii*	USNM 1421206	M	China	South China Sea, FAO Fishing Area 61	MK270942	MK271044	MK281244
*Monomiahaanii*	USNM 1420827	F	Taiwan	Daxi Fishery Port	MK270933	MK271031	MK281231
*Monomiahaanii*	USNM 1420828	M	Taiwan	Daxi Fishery Port	MK270955	MK271032	MK281232
*Monomiahaanii*	UF 29509	F	Taiwan	Daxi Fishery Port	MK270930	MK271026	MK281227
*Monomiahaanii*	UF 29511	F	Taiwan	Daxi Fishery Port	MK270931	MK271027	MK281228
*Monomiahaanii*	UF 29512	F	Taiwan	Daxi Fishery Port	MK270932	MK271028	—
*Monomiahaanii*	ZRC 1998.0186	M	Taiwan	Daxi Fishery Port	MK270947	MK271059	MK281254
*Monomiahaanii*	MNHN-IU-2014-10086		Vietnam	Của Bé Fishing Port	—	KY524463 ^1^	—
*Monomiahaanii*	UO 12J-Vn12		Vietnam	Của Bé Fishing Port	—	KY524464 ^1^	—
*Monomiahaanii*	ZMMU Ma 3368		Vietnam	Của Bé Fishing Port	—	—	JX398094 ^2^
*Monomiaargentata*	MNHN-IU-2014-10076		Vietnam	Của Bé Fishing Port	—	KY524480 ^1^	—
*Monomiaargentata*	MNHN-IU-2014-10075		Vietnam	Của Bé Fishing Port	—	KY524479 ^1^	—
*Monomiaargentata*	IOM		Vietnam	Của Bé Fishing Port	—	KY524478 ^1^	—
*Monomialucida*	ZRC 2016.0150	M	Vanuatu	South Pacific Ocean	MK270965	MK271061	—
*Monomialucida*	ZMMU Ma 3365		Vietnam	Của Bé Fishing Port	—	—	JX398096 ^2^
*Monomialucida*	NHMUK 2017.402		Vietnam	Của Bé Fishing Port	—	MG563792 ^3^	—
*Monomialucida*	MNHN-IU-2014-10083		Vietnam	Của Bé Fishing Port	—	MG563793 ^3^	—
*Monomialucida*	MNHN-IU-2014-10085		Vietnam	Của Bé Fishing Port	—	MG563794 ^3^	—
*Monomiapetrea*	UF 188		Guam	Tepungan Channel	—	KT365606 ^4^	KT365743 ^4^
**Outgroup Taxa**							
*Portunussanguinolentus*	ZRC 2016.0146	M	India	Pazhayar Fish Landing	MK270966	MK271057	—
*Portunuspelagicus*	ZRC 2016.0147	M	India	Porto Novo	MK270967	MK271058	MK281258

Sequences mined from GenBank are attributed to ^1^Koch and Ďuriš (2018), ^2^Spiridonov et al. (2014), ^3^Koch et al. (2017), and ^4^Evans (2018). *Holotype of Portunuspseudoargentatus Stephenson, 1961

### Commercial products

Four cans (454 g each) of pasteurized lump crabmeat labeled as “Portunushaanii” were purchased from grocery stores in Maryland and Virginia in 2016 and 2017. Portions of 10 lumps (i.e., single piece of crabmeat reasonably expected to be from an individual crab), five from the top and five from the bottom, from each tub were sampled for DNA extraction (N=40). The DNA barcode region of the cytochrome oxidase subunit I (COI) was amplified and sequenced from samples following the methods described below.

### Molecular methods

Genomic DNA was extracted from muscle tissue dissected from ethanol preserved or fresh specimens using the DNeasy Tissue Kit (Qiagen) according to the manufacturer’s animal tissue protocol. Portions of three mitochondrial genes were amplified: a 658 bp barcode region of the cytochrome c oxidase I gene using the primers JgLCO1490 and JgHCO2189 (Geller et al. 2013), a 531 bp region of the 16S ribosomal gene using the primers 16S-ar and 16S-br (Palumbi 1996), and a 375 bp region of the 12S ribosomal gene using the primers 12Sf (Mokady et al. 1994) and 12S1R (Shull et al. 2005). PCR was carried out for 35 cycles with an annealing temperature of 48 °C for COI and 52 °C for 12S and 16S using Promega GoTaq G2 hot start master mix (Promega M7432). PCR products were visualized by agarose gel electrophoresis (1.5% agarose) and purified with ExoSAP-IT (Affymetrix) prior to sequencing. Sequencing reactions were performed using 1 μL of purified PCR product in a 10 μL reaction containing 0.5 μL primer, 1.75 μL Big Dye buffer and 0.5 μL Big Dye (Life Technologies).

Geneious 9.1.7 (Biomatters) was used to visualize, trim, edit, and assemble contigs from forward and reverse sequences. All PCR, sequencing, and analytics were carried out at the Laboratories of Analytical Biology at USNM. Sequences have been deposited in GenBank (NCBI) with accession numbers listed in Table 1.

Partial sequences for each locus were also amplified from *Portunuspelagicus* (ZRC 2016.0147) and *P.sanguinolentus* (ZRC 2016.0146) to serve as outgroup taxa. Multiple sequence alignments were generated using the L-INS-i alignment strategy in MAFFT version 7 (Katoh and Standley 2013). The aligned sequences were then concatenated using Sequence Matrix (Vaidya et al. 2011). In the concatenated data set, positions 1–380 are 12S, 381–914 are 16S, and positions 915–1572 are COI.

A best-fit model of nucleotide sequence evolution compatible with MrBayes and partitioning arrangement for each locus was determined using Partition Finder 2 (Lanfear et al. 2016) with the greedy algorithm selected (Lanfear et al. 2012). The GTR+I+G model was chosen for all three loci. Phylogenetic analyses were performed on the concatenated dataset using maximum likelihood (ML) with RAxML (Stamatakis 2006) and Bayesian Inference (BI) performed with MrBayes 3.1.2 (Ronquist and Huelsenbeck 2003) on the FDA’s Raven2 high performance computing cluster. ML options for RAxML included the GTRCAT model of nucleotide evolution (-m), rapid bootstrap analysis, and search for best-scoring ML tree (-f a), and 1000 bootstrap replicates. BI analysis was carried out for 10 million generations with two independent runs, each with four chains, and with trees sampled every 1000th generation. Model parameters (tratio, statefreq, shape, pinvar) were unlinked among partitions, and the rate prior (prset ratepr) was set to “variable.” To calculate posterior probabilities, a “burn-in” of 25% of the total trees sampled per run adequately removed trees prior to convergence.

In addition to the concatenated dataset, a COI-only dataset which incorporated sequences from GenBank and BOLD was analysed to identify the species of crab found in four cans of pasteurized lump crabmeat labelled as “*Portunushaanii*.” For visualization purposes, a neighbour joining tree of the 658bp alignment was built using the Jukes-Cantor model in the Geneious Tree Builder. Patristic and K2P distances were calculated for each alignment using MEGA7 (Kumar et al. 2016).

## Results

### Molecular phylogenetics

Molecular phylogenetic analyses of the concatenated dataset of three mitochondrial loci show that there is a well-supported (98/1) separation between *M.gladiator* and *M.haanii*. *Monomiapetrea* (Alcock, 1899) (UF188; KT365743, KT365606) is a strongly supported sister to both (95/1) (Fig. 5). Analysis also confirms that voucher specimens purchased at the Daxi Fishery Port in northern Taiwan as well as those supplied by U.S. seafood importer, Newport International, both under the name “*P.haanii*,” are the same species as the specimen of “*M.pseudoargentata*” (ZMMU 3368; JX398094) from Spiridonov et al. (2014). Furthermore, our specimens with color patterns similar to “*M.haanii*” in Chertoprud et al. (2012) (= *M.gladiator*ZMMU 3366 in Spiridonov et al. 2014; JX398095) are the same species: *M.gladiators. str.* (Fig. 5). Included in the clade of *M.haanii* is the holotype of *Portunuspseudoargentatus* (WAM-C7056). The topology of the ML phylogram is congruent with the morphological findings.

DNA barcode sequence analyses for species identification of products confirm that crabmeat sold as “*Portunushaanii*” is indeed what we have identified herein as *Monomiahaaniis. str.* (Fig. 6). The mean K2P distance between reference *M.haanii* and the 40 product samples is 0.72% compared to the 7.85% between *M.gladiator* and product samples. Similarly, COI sequences from *M.haanii* and *M.gladiator* reference samples have a mean K2P distance of 7.74% (Table 2) and is consistent with congeneric divergences observed in other decapods (Costa et al. 2007).

**Table 2.** Mean K2P distances between specimens genetically identified as *M.gladiator*, *M.haanii*, and commercial products calculated from a neighbor-joining distance tree built in Geneious.

	***Monomiagladiator***	***Monomiahaanii***
***Monomiahaanii***	7.74%	
**Commercial Products**	7.85%	0.72%

## Taxonomic accounts

### Portunoidea Rafinesque, 1815

**Portunidae Rafinesque, 1815**

**Portuninae Rafinesque, 1815**

***Monomia* Gistel, 1848**

**Type species.***Portunusgladiator* Fabricius, 1798, type species of *Amphitrite* De Haan, 1833, by subsequent designation (Miers, 1886); pre-occupied by *Amphitrite* Müller, 1771 [Polychaeta]; *Monomia* Gistel, 1848, replacement name for *Amphitrite* De Haan, 1833.

#### Monomia gladiator

Taxon classificationAnimaliaDecapodaPortunidae

s. str. (Fabricius, 1798)

**Figure 1.**
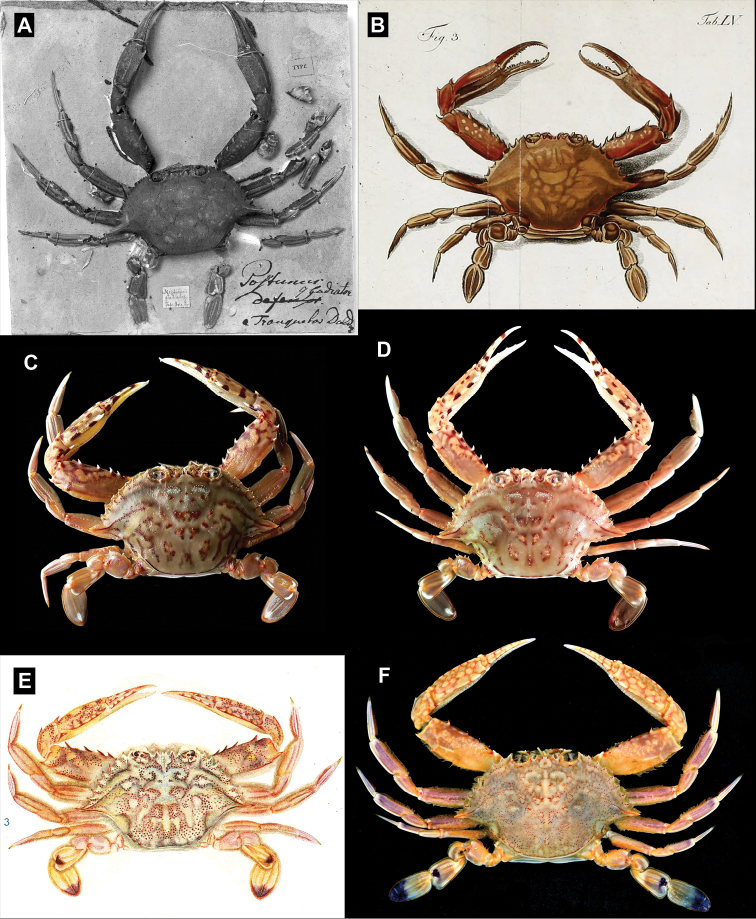
Dorsal habitus of **A** lectotype of *Portunusgladiator* Fabricius, 1798, deposited in Copenhagen Museum (ZMUC-Cru 4705) **B***Cancer menestho* Herbst, 1803 (= *Monomiagladiator*), probably from Indian Ocean (color print from Herbst, 1803: pl. 55 fig. 3 **C***Monomiagladiator* (Fabricius, 1798), fresh colouration, Phuket, Thailand (not collected), photo by Rueangrit Promdam **D***Monomiagladiator* (Fabricius, 1798), fresh coloration, Jeppiar, Tamil Nadu, India (ZRC), photograph by PKL Ng; **E** “Neptunus (Amphitrite) gladiator” [sic] (=*Monomiahaanii*) from Sagami Bay, Japan (color print from Sakai, 1939: pl. 47 fig. 3) **F***Monomiahaanii* (Stimpson, 1858), fresh colouration, South China Sea (USNM 1421161) shipped frozen by US seafood importer.

Figs 1A–D , 3A–C , 4A–D

Portunus gladiator Fabricius, 1798: 368; Latreille 1825: 189; Crosnier 1962 (in part): 51, figs 72, 76, 78, 82, 83, pl. 3 fig. 2; Stephenson and Rees 1967a: 14; 1967b: 25; 1968: 293 (in part); Stephenson 1972a: 16, 39 (in part); 1972b: 135 (in part); Bhadra 1998: 410; Dev Roy and Bhadra 2005: 425; 2011: 147.
Cancer menestho Herbst, 1803: 34, pl. 55 fig. 3.
Lupea gladiator , H. Milne Edwards 1834: 456.
Neptunus gladiator , A. Milne-Edwards 1861: 330; Richters 1880: 152; Müller 1887: 475; De Man 1888: 69; Henderson 1893: 367.
Neptunus (Amphitrite) gladiator , Miers, 1886: 177; Alcock 1899: 35, 36; Laurie 1906: 412.
Callinectes gladiator , Stebbing 1915: 58. Non Callinectesgladiator Benedict, 1893 (fide Stephenson and Cook 1973).
Monomia gladiator , Barnard 1950: 156; Fourmanoir 1954: 9; Spiridonov et al. 2014: table 1; Trivedi et al. 2018: 66, table 1.
Portunus (Monomia) gladiator , Jeyabaskaran et al. 2000: 51, pl. 36c; Biju Kumar et al. 2007: 286; Ng et al. 2008: 151, 156, 157 (list and discussion).
Portunus (Monomia) gladiator [sic], Krishnamoorthy 2009: 6 (list).
Portunus haanii , Stephenson and Cook 1973: 429 (in part), figs 6A–E, 7A–E, 8A–E, 9A, 10A, C, D, G; Stephenson 1975: 178. Non Amphitritehaanii Stimpson, 1858.
Monomia haanii , Chertoprud et al. 2012: 314, pl. 51 fig. G. Non Amphitritehaanii Stimpson, 1859.
Non Cancergladiator Fabricius, 1793: 449 (= Portunussanguinolentus (Herbst, 1783), fide Latreille 1825).
Non Portunus (Amphitrite) gladiator, De Haan 1833: 65; 1835: pl. 18 fig. 1 (= Portunusorbitosinus Rathbun, 1911).
Non Portunusgladiator, Stephenson and Campbell 1959: 110, Figs 2J, 3J, pl. 3 fig. 2, pl. 4 fig. J, pl. 5 fig. J (= Portunusaustraliensis Stephenson and Cook, 1973).

##### Material examined.

INDIA: ZRC.2016.0145, 2 males, 1 female, Pazhayar Fish Landing, Nagapattinam District, Tamil Nadu, coll. NK Ng et al., 17 Sep. 2011; ZRC.2016.0149, 1 female, sandy beach, Pondicherry, Union Territory of Puducherry; ZRC.2018.1189, 5 males, Jeppiar Fishing Port, Muttom, Tamil Nadu, coll. PKL Ng et al., 19 Sep. 2016; USNM127069, 1 male, SW of Mumbai, IIOE Anton Bruun, 14 Nov. 1963.

AUSTRALIA: WAM-C26459, 2 males, Dampier Archipelago, Cape Brugieres, Western Australia, coll. Slack-Smith and Hewitt, 17 Jul. 1999; WAM-C61155, 2 females, WAM-C61156, 1 female, Pilbara Shelf, Western Australia, coll. E Morello et al. (CSIRO Pilbara Survey), 13 Jun. 2013.

PENINSULAR MALAYSIA: ZRC.2000.1308, 4 males, Perhentian, coll. anon., 16 May 1976.

MYANMAR: ZRC.2016.0030, 1 female, Ayeyarwady Delta, coll. EAF-Nansen Project (Myanmar cruise), 19 May 2015; ZRC.2016.0034, 1 male, Tanintharyi Coast, coll. EAF-Nansen Project (Myanmar cruise), 26 May 2015.

SINGAPORE: ZRC.1965.10.22.1-2, 1 male, 1 female, Siglap, coll. M.W.F. Tweedie, Jul. 1933; ZRC.1984.338-348, 4 males, 7 females, Horsburgh Lighthouse, South China Sea near Singapore, coll. H Huat, 15 Dec. 1982; ZRC.1984.5451-5453, 3 males, Tuas fishery port, coll. WM Lee, 25 Sep. 1982.

THAILAND: USNM127068, 2 females, Andaman Sea, north of Phuket, IIOE Anton Bruun R/V, 31 July. 1963; ZRC.2000.0779, 3 males, 13 females, Phuket, Pichai Fish Port, coll. NK Ng et al., 17–20 Jan. 2000; ZRC.2000.0842, 2 males, Phuket, Pichai Fish Port (Andaman Sea), coll. PKL Ng et al., 3–6 May 2000; ZRC.2002.0297, 4 males, 1 female, Phuket, Pichai Fish Port, coll. JCY. Lai, 2–3 Sep. 2001; ZRC.2002.0298, 3 males, 1 female, Phuket, Pichai Fish Port, coll. JCY Lai, 2–3 Sep. 2001; ZRC.2003.0114, male, Pattani Fishing Port, Pattani Province; ZRC.2003.0197, 1 male, Saiburi Crab Landing, Pattani Province, coll. Z Jaafar et al., 8 Jun. 2003.

##### Diagnosis.

Carapace (Fig. 1A–D) transversally hexagonal, CW/CL ratio 1.79–1.83, with dorsal surface, except patches of granules, densely covered by short tomentum. Regions moderately defined; with discrete patches of granules on gastric, branchial, cardiac and intestinal regions. Front subdivided into four teeth with rounded apices, median pair distinctly smaller than lateral; median sulcus between teeth continuing ventrally to triangular projection appressed to median epistomial tooth. Epistome well defined, median tooth projecting beyond front. Supraorbital margin finely granulate, with two distinct notches; inner orbital angle tooth-like, with glabrous ventromesial ledge. Infraorbital margin with deep, V-shaped notch laterally; in antero-ventral view, mesial part of infraorbital margin granulate, ventrally a large triangular tooth projecting anteriorly, visible from dorsal view. Anterolateral margin armed with nine teeth, including external orbital angle, with granulate margins; first tooth larger than following teeth except 9^th^, with straight outer margin; 2^nd^–8^th^ subequal in size, sharp, projecting outward, slightly curved anteriorly; 9^th^ tooth largest, projecting laterally; just underneath anterolateral margin is thick coat of long soft setae which sometimes obscures teeth. Posterolateral margin concave, posterolateral angle rounded; posterior margin lined with small granules, straight to slightly convex; posterior margin with ventrally directed, smooth flange, lateral extremities of which coincide with posterolateral angle of carapace, each armed with small, lamelliform tooth.

**Figure 2.**
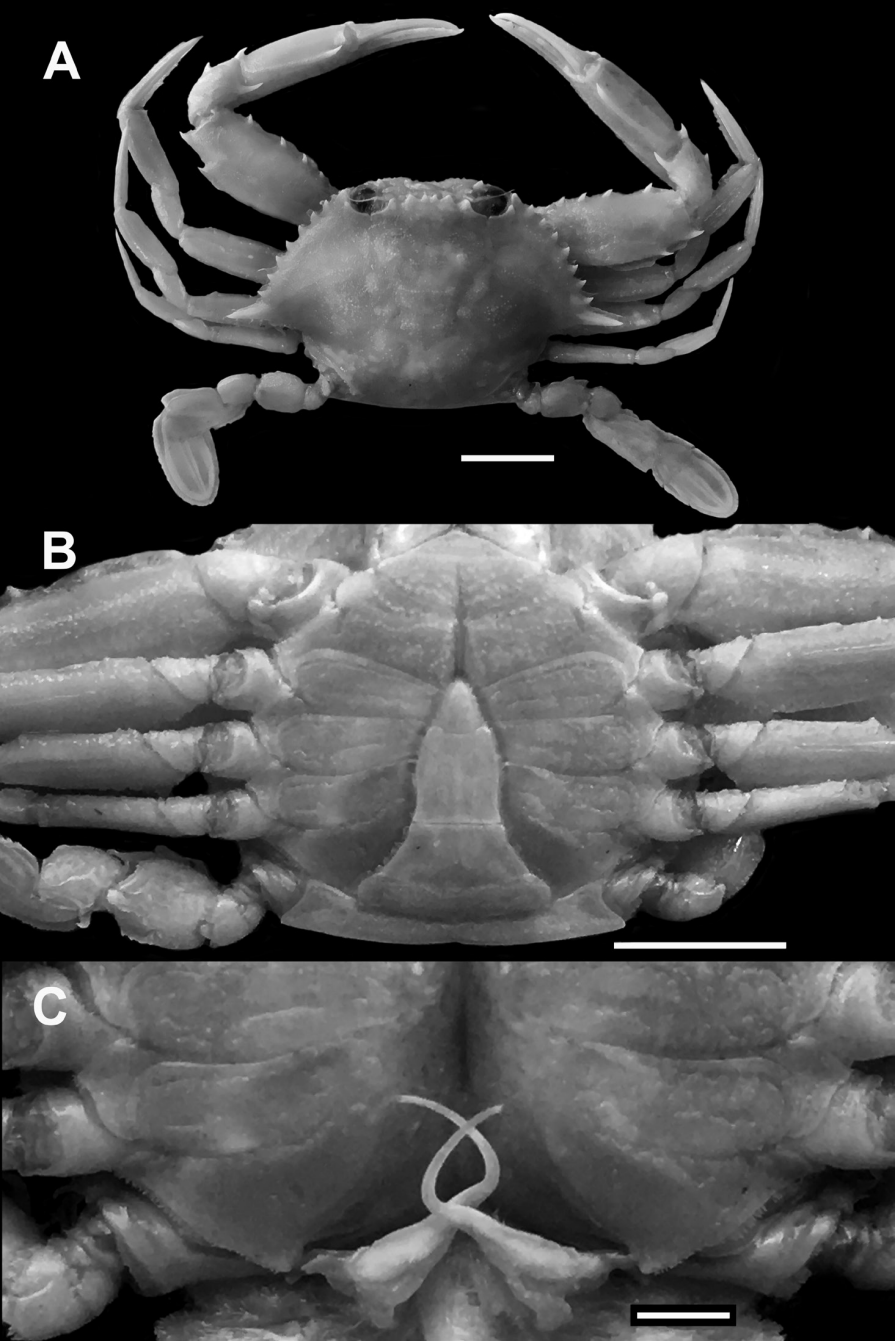
*Monomiahaanii* (Stimpson, 1858), lectotype, male (RMNH 379), Japan. **A** dorsal habitus **B** thoracic sternum and pleon, ventral view **C** sterno-pleonal cavity and G1s, ventral view. Photographs by CHIJ Fransen (RMNH). Scale bars: A, B 10 mm; C 3 mm.

Basal article of antennule completely filling antennular fossa, subsequent two articles slender. Basal article of antenna short, with broad, lateral projection entering but not obstructing orbital hiatus; flagellum long, exceeding well beyond orbit. Eyes with well-developed corneas, short, thick peduncles. Proepistome well developed, anterior tip with projecting conical tooth; epistome not extensively projecting posteriorly. Endostome with well-developed lateral ridges.

Third maxillipeds finely granulate on ischium, merus and exopod, setose on external surfaces, extensively pilose on mesial margins; ischium longer than wide, subrectangular, with deep, submesial sulcus; merus longer than wide, rhomboidal, anterolateral angle strongly projecting laterally; palp articles subcylindrical. Exopod stout, with subdistal triangular projection on inner medial border; flagellum well developed.

**Figure 3.**
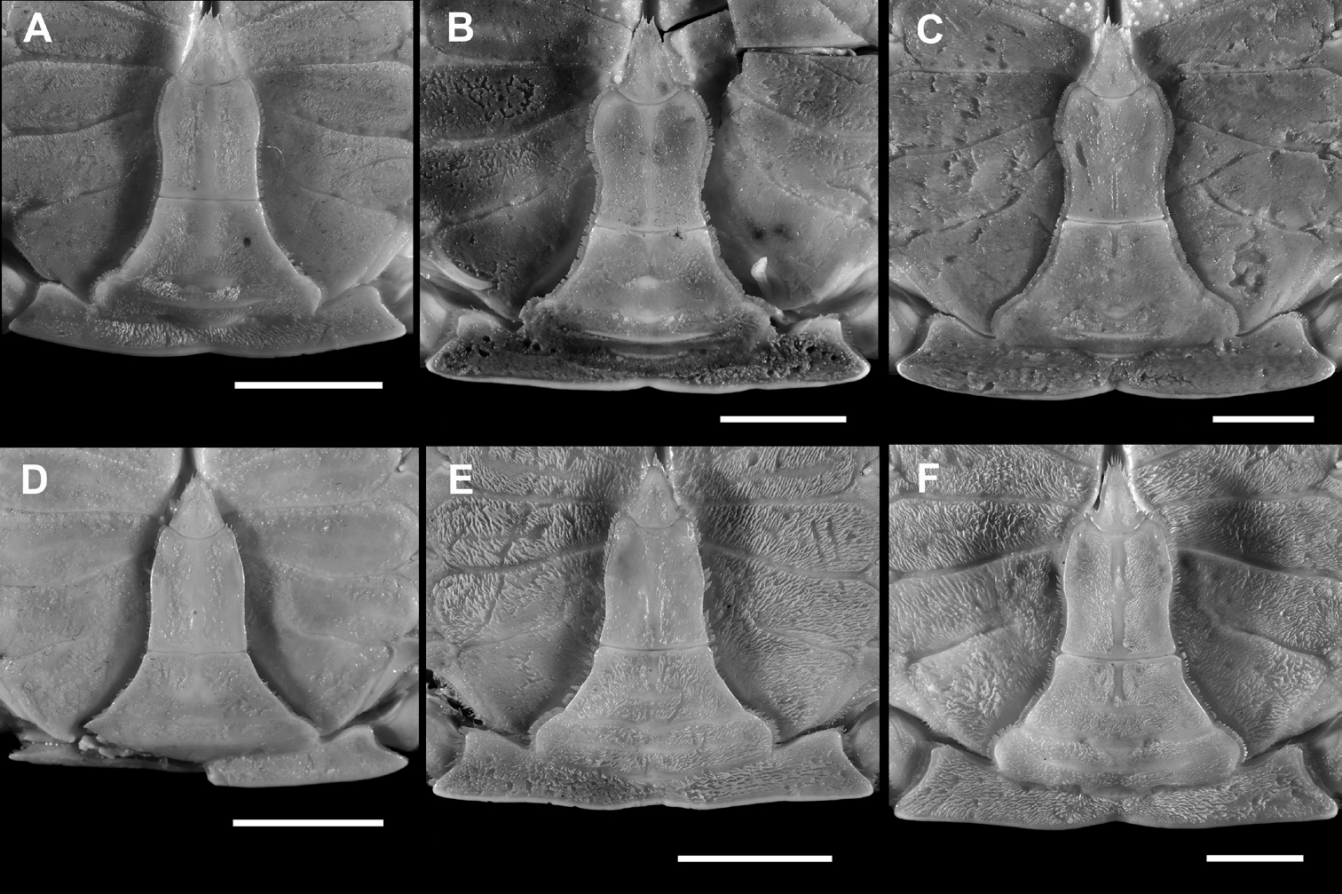
Male thoracic sternum and pleon. **A–C***Monomiagladiator* (Fabricius, 1798), **A**ZRC 2018.1189, Jeppiar, Tamil Nadu, India **B**ZRC 2016.0145, Pazhayar Tamil Nadu, India; C) ZRC 2000.0842, Phuket, Thailand. **D–F***Monomiahaanii* (Stimpson, 1858) **D**WAM-C7506, holotype of *Portunuspseudoargentatus* Stephenson, 1961, Abrolhos Is., Western Australia **E**ZRC 1999.0084, Boso Peninsula, Japan **F**ZRC 2016.0408, Daxi Fishery Port, Taiwan. Scale bar: 10 mm.

Male thoracic sternites covered with thick tomentum, thickest on exposed surfaces of sternites 5–8 (Fig. 3A–C); sternites 1–4 distinctly granulate, with granules becoming larger, coarser in large specimens; sternites 1, 2 fused, separated from sternite 3 by straight suture; sternites 3, 4 almost completely fused except for remnants of suture at lateral extremities, replaced mesially by smooth, setose groove; sternite 4 with narrow median groove on exposed surface, continuing posteriorly into sterno-pleonal cavity. Sutures 4/5, 5/6, and 6/7 present on exposed surface of thoracic sternum but interrupted within sterno-pleonal cavity; suture 7/8 present on most of exposed thoracic sternal surface, but disappearing just before sternopleonal cavity. Median longitudinal line evident at level of sternites 6, 7, 8, absent elsewhere. Press-button tubercle of sternopleonal locking mechanism located on posteromesial projection of sternite 5.

Chelipeds (P1), long, robust, surfaces tomentose; slightly heterochelous, major chela usually with modified cutting/crushing tooth proximally on cutting margin of dactylus. Merus long, with 4, sometimes 5, curved spines along flexor margin, and 2 distal spines on extensor margin; both margins densely setose. Carpus with sharp spine on inner angle, and flattened spine on external surface continuing as a strong carina, with additional, shorter carina above it. Dorsal surface of palm (propodus) with two straight, longitudinal granular crests, inner one distally ending distally in strong spine; small proximal spiniform tooth at articulation with carpus; two additional, curved granular crests on external surface of palm, first ending at level of articulation with dactylus, second, lower, ending near gape, creating cristate, proximo-ventral margin of palm; inner surface of palm with two wide, distinct rows of granules. Fingers generally straight except for curved, pointed tips; subequal in length to palm; with two granulate crests each on external and internal surfaces; lowest carina on fixed finger extending into palm; numerous teeth on cutting margins, arranged in groups so that each group has large central tooth flanked by smaller teeth of decreasing size, giving the cutting margins appearance of having three or more denticulate, triangular lobes.

First to third ambulatory legs (P2–P4), long, slender; decreasing in length and size, with P2 largest, P4 smallest; flexor margins of meri, carpi, propodi and dactyli heavily setose. Fifth ambulatory (natatory) leg (P5) with quadrate merus, pentagonal carpus, flat, subrectangular propodus, and flat, oval dactylus; margins of articles regularly setose; propodus with four raised glabrous longitudinal bands, including flexor and extensor margins, interspersed with tomentum; dactylus with five raised glabrous bands, including flexor and extensor margins, interspersed with tomentum, distal third with low median crest continuing proximally as narrow tomentose stripe; in fresh specimens, P5 propodus with white band on postero-distal margin, no purple spot, P5 dactylus with small white spot on distal end.

Male pleon (Fig. 3A–C) ‘inverted T’-shaped, external surfaces mostly tomentose, 3^rd^–5^th^ pleomeres fused. First pleomere very thin, less wide, mostly obscured by flange of posterior margin of carapace. Second pleomere much wider than first, lateral edges resting on P5 coxa, with prominent transverse keel running along entire width. Third pleomere widest, formed like a wedge, visible from both dorsal and ventral view; strong transverse crest somewhat forming posterior margin of cephalothorax, with shallow notch medially, posterolateral angles sharp, acute; sulcus between fused 3^rd^ and 4^th^ pleomere moderately deep, glabrous. Fourth pleomere subrectangular, wider than long, lateral margins convex, central region with low transverse crest. Fifth pleomere subtrapezoidal, basal margin wider than anterior. Sixth pleomere subrectangular; median length about 1.2 times maximum width; width at maximum lateral convexity greater than basal width; anterior margin concave, basal margin straight, lateral margins convex anteriorly, concave posteriorly. Telson subtriangular, apex rounded, lateral margins slightly concave, basal margin convex; median length 1.2 times basal width, with rounded tip, broadly rounded posterior margin.

**Figure 4.**
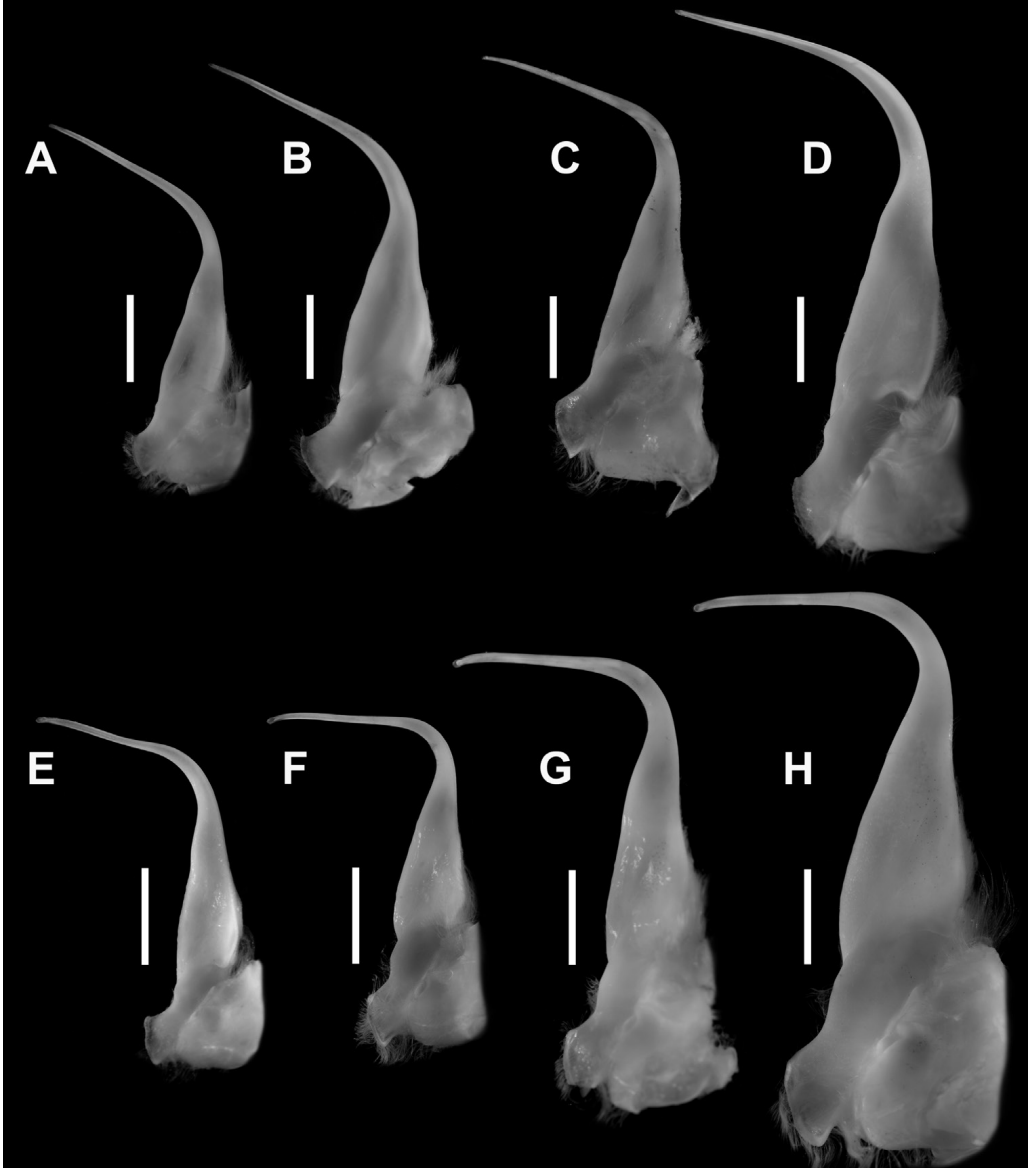
Left G1, sternal view (except D and E). **A–D***Monomiagladiator* (Fabricius, 1798): A) ZRC 2018.1189, Jeppiar, Tamil Nadu, India **B**ZRC 2016.0034, Tanintharyi coast, Myanmar **C**ZRC 2016.0145, Pazhayar, Tamil Nadu, India **D**ZRC 2000.0842, Phuket, Thailand (flipped right G1). **E–H***Monomiahaanii* (Stimpson, 1858) **E**WAM-C7506, holotype of *Portunuspseudoargentatus* Stephenson, 1961, Abrolhos Is., Western Australia (flipped right G1) **F**ZRC 1999.0084, Boso Peninsula, Japan **G**ZRC 2016.0408, smaller male, Daxi Fishery Port, Taiwan **H**ZRC 2016.0408, larger male, Daxi Fishery Port, Taiwan. Scale bar: 3 mm.

G1 (Figs 4A–D) with proximal half relatively wide, somewhat flattened, strongly bent medially, by at least 45° but much less than 90°, distal half very slender, filiform; apically slightly recurved, rounded; distal tip much narrowed to small unarmed aperture. G2 about half length of G1, slender, distal tip minutely bilobed. Penis long, slender, uncalcified; emerging from sternocoxal condyle of P5.

##### Remarks.

Following the recognition of *Monomia* Gistel, 1848, as a genus distinct from *Portunus* Weber, 1795 (see Mantelatto and Robles 2007; Mantelatto et al. 2009; Schubart and Reuschel 2009; Chertoprud et al. 2012; Spiridonov et al. 2014), the taxonomy of its type species, *Monomiagladiator* (Fabricius, 1798), needs to be assessed.

Firstly, there are five specimens identified as syntypes of *Portunusgladiator* Fabricius, 1798, in the Zoological Museum of the University of Copenhagen (ZMUC) (see http://www.zmuc.dk/inverweb/invertebrater/Crustacea%20databases/Fabricius%20collection.htm). All are dry specimens with the catalog numbers ZMUC-CRU 4704 through 4708 (see Ng et al. 2008). Examination of their photographs (available online) show that one of them is clearly not conspecific nor even congeneric (viz. ZMUC-CRU 4707). Accompanying this specimen in the photograph is a handwritten label identifying it as “*Achelous Whitei* A. M. Edw.,” a name now considered a junior synonym of *Lupocycloporusgracilimanus* (Stimpson, 1858) (viz. Ng et al. 2008), a widespread species known from several localities in the Indo-West Pacific region; and a check with the available literature (e.g., Stimpson, 1907: pl. 10 fig. 3) confirms this identification. To stabilize the taxonomy of *Portunusgladiator* Fabricius, 1798, one of the other four syntypes, a male ZMUC-CRU 4705 (Fig. 1A), is hereby designated as the lectotype, and the other three specimens (ZMUC-CRU 4704, 4706 and 4708) become paralectotypes.

Secondly, we agree with Ng et al. (2008) that there is no secondary homonymy between *Portunusgladiator* Fabricius, 1798, and *Cancer gladiator* Fabricius, 1793, because the two species were originally described in different genera, and the name “*Cancer gladiator* Fabricius, 1793” is no longer available by virtue of its synonymization under *Portunussanguinolentus* (Herbst, 1783) (viz. Latreille 1825; Stephenson and Cook 1973). Although Chertoprud et al. (2012) cite the use of the name “*Portunusgladiator* (Fabricius, 1793)” by Stephenson and Cook (1973) as meeting the requirement of Article 59.1 of the Code, we view this as a misinterpretation of the Code’s provisions for homonymy. Furthermore, with the recognition of *Monomia* Gistel, 1848, as a full genus, thus absolutely eliminating any reservations concerning secondary homonymy, the species name *Monomiagladiator* (Fabricius, 1798) is, therefore, valid.

Thirdly, there is the matter of the confusion between *M.gladiator* and *M.haanii*. Stephenson and Cook (1973) proposed *Amphitritehaanii* Stimpson, 1858, as a replacement name for *P.gladiator* Fabricius, 1798, and this has led to the current confusion between the two names. As the following discussion will show, it is evident that *Monomiagladiator* and *M.haanii* (Stimpson, 1858) are distinct and valid species, and, therefore, the latter cannot be used as a replacement name for the former.

*Monomiagladiator* differs from *M.haanii* primarily in these three morphological characters: (1) in the fresh specimens of *M.gladiator*, there is a white band on the postero-distal margin of the P5 propodus, but no purple spot, and a small white spot on the distal tip of the P5 dactylus (Fig. 1C, D; also Chertoprud et al. 2012: pl. 51 fig. G) (vs. in *M.haanii*, there is a large purple spot on the distal tip of the P5 propodus, and the distal one-third of the P5 dactylus is colored purple; Fig. 1E, F; also Sakai, 1939: pl. 47 fig. 3; 1965: pl. 57, fig. 1; Chertoprud et al. 2012: pl. 51 fig. H); (2) the anterolateral margins of the male 6^th^ pleomere are more flared out and rounded in *M.gladiator*, and at their widest extent exceed the basal width of the 6^th^ pleomere (Fig. 3A–C; also Stephenson and Cook, 1973: fig.10A) (vs. straight, convergent anteriorly, and separated from concave posterolateral margin by an angular convexity in *M.haanii*, widest at base, Figs 2B, 3D–F); and (3) the G1 is bent at an angle of about 45° at mid-length and the distal tip is slender and relatively narrower in *M.gladiator* (Fig. 4A–D) (vs. G1 bent at or almost at 90°, and distal tip is somewhat wider than the immediate subdistal region in *M.haanii* (Figs 2C, 3E–H). Aside from these are some minor differences; for instance, the mesial part of the infraorbital margin of *M.gladiator* is granulate and there is no tooth on the mesial end as it abuts the basal article of the antenna, there is, however, a large tooth immediately ventral to this margin, which projects outward and is visible from dorsal view as if it were part of the infraorbital margin. In *M.haanii*, the mesial end of the infraorbital margin has a large tooth which abuts against the basal article of the antenna. Also, the spines on the flexor margin of the P1 merus of *M.gladiator* tend to be more projecting and recurved than those of *M.haanii*, which are relatively smaller and less curved. Examination of available fresh-color photographs (viz. Chertoprud et al. 2012: pl. 51 Figs G, H) also show that these meral spines tend to be entirely white in *M.gladiator* while they are red at the base and white at the tips in *M.haanii*.

Furthermore, the molecular phylogenetic analysis corroborates the morphological evidence, clearly showing two distinct and well-supported clades corresponding to the two species. Specimens identified as *M.gladiator* based on the characters described above, including a topotypic specimen from India (ZRC.2016.0149), form a well-supported clade distinct from another clade containing specimens with the morphological attributes of *M.haanii*, which also includes a topotypic specimen from Japan (ZRC.2000.0084). Furthermore, the same molecular phylogenetic analysis shows that the specimen referred to by Chertoprud et al. (2012) as “*Monomiahaanii*” (JX398095) falls within the *M.gladiator* clade, whereas the specimen referred to by Chertoprud et al. (2012) as “*Monomiapseudoargentata*” (JX398094) falls within the *M.haanii* clade. These specimens should now be identified as *M.gladiator* and *M.haanii*, respectively.

Finally, *Lupeagladiator* H. Milne Edwards, 1834 (Indian Ocean), is re-included in the synonymy of *Monomiagladiator* (Fabricius, 1898), and *Cancer menestho* Herbst, 1803 (probably from Indian Ocean) is hereby considered a junior subjective synonym of *Monomiagladiator* (Fabricius, 1798). We believe that Stephenson and Cook (1973) erred in removing these two names from the synonymy of *gladiatorsensu* Fabricius, 1798. In the case of H. Milne Edwards’ specimen, the error is due simply to their conflation of *haanii* with *gladiator*. In the case of Herbst’s species, we disagree with them that the illustration of *Cancer menestho* does not show 2 spines on the posterior margin of the cheliped merus. What they call a “non-protruding” spine is an artifact of perspective. We have seen in our photographs of *M.gladiator*, that this second spine can appear non-protruding when the merus is viewed from directly above and if the marginal setae obscure its entire outline. Once this so-called difference is eliminated, there is no compelling reason why *C.menestho* should also not be treated as a synonym of *M.gladiator*.

#### Monomia haanii

Taxon classificationAnimaliaDecapodaPortunidae

s. str. (Stimpson, 1858)

Figs 1E, F , 2 , 3D–F , 4E–H

Portunus (Amphitrite) gladiator : De Haan 1833: 39; 1835: pl. 1 fig. 5, pl. A. Non Portunusgladiator Fabricius, 1798.
Amphitrite haanii Stimpson, 1858: 38; 1907: 79; Ng et al. 2008: 151 (synonymy), 156 (discussion).
Neptunus (Amphitrite) gladiator : Ortmann 1893: 73; Lanchester 1902: 544; Parisi 1916: 173; Balss 1922: 107; Sakai 1934: 303; 1936: 129, pl. 36 fig. 3; 1939: 390, fig. 5a, pl. 47 fig. 3; Shen 1937: 101, fig. 2; Lin 1949: 19. Non Portunusgladiator Fabricius, 1798.
Portunus gladiator : Rathbun 1902: 26, Miyake 1961: 172; Sakai 1965: 118, pl. 57 fig. 1; 1976: 341, fig 180a, pl. 120 fig. 1; Stephenson and Rees 1967: 24; 1968: 293; Takeda and Miyake 1968: 551; Stephenson 1972a: 16, 39; 1972b: 135; Ko and Lee 2013: 40, pl.-fig. 34. Non Portunusgladiator Fabricius, 1798.
Portunus (Achelous) gladiator ; Rathbun 1910: 36 (from Gulf of Siam). Non Portunusgladiator Fabricius, 1798.
Portunus pseudoargentatus Stephenson, 1961: 109, Figs 2A, 3F, pl. 2 fig. 4, pl. 4 fig. F, pl. 5 fig. D; Stephenson and Rees 1967b: 25; 1968: 294; Yang and Dai1994: 137, fig. 13.
Portunus haanii : Stephenson and Cook 1973 (in part): 429, figs 6F–H, 7F–H, 8F–H, 9B; Dai and Yang 1991: 223, fig. 120(2), pl. 27(4); Moosa 1996: 521; Apel and Spiridonov 1998 (in part): 291; Ng et al. 2001: 16 (list).
Portunus (Monomia) gladiator : Sakai 1976: 341, fig. 180, pl.120 fig. 1; Kim and Chang 1985: 52. Not Portunusgladiator Fabricius, 1798.
Portunus haanii : Miyake 1983: 85, pl. 29 fig. 2; Takeda 1989: 152
Portunus (Monomia) haanii Yamaguchi & Baba, 1993: 396, figs 137A–C.
Monomia pseudoargentata : Chertoprud et al. 2012: 315, pl. 51 fig. H; Spiridonov et al. 2014: 412, 427, fig. 3I, tab. 1.
Monomia haanii : Ng et al., 2017: 68 (list).
Not Amphitritemedia Stimpson, 1858: 39; 1907: 79, pl. 10 fig. 1.
Not Monomiahaanii, Chertoprud et al. 2012: 314, pl. 51 fig. G (=Monomiagladiator (Fabricius, 1798)).

##### Material examined.

JAPAN: RMNH 379, lectotype, male, Japan, coll. P.F. von Siebold, 1823–1829 (photographs only); ZRC.1999.0084, 1 male, 1 female, off Hota, Uchibo, coast of Boso Peninsula, coll. T Komai, 22 Aug. 1997; USNM5255, 4 male, 1 female, coll. FC Dale and PL Jouy, Palos R/V; USNM26254, 1 male, 1 female, off Wakanoura, coll. DS Jordan and JO Snyder, 1900; USNM45882, 1 female, off Wakanoura, Kishu; USNM54519, 1 female, Yamagata Prefecture, coll. M Sasaki, Aug. 1917; USNM72540, 2 males, Enoshima, Bay of Jeddo, coll. ES Morse; USNM60250, 1 male, Toyama Bay, coll. M Sasaki, 1925; USNM112423, 1 male, Shimizu, Sugura, Albatross R/V, 14 Oct. 1906.

AUSTRALIA: WAM-C7506, 1 male (holotype of *Portunuspseudoargentatus* Stephenson, 1961), Abrolhos Islands, Western Australia, coll. RW George, 11 May 1960; WAM-C34767, 1 female, Exmouth Gulf, Western Australia , coll. S Morrison and P Unsworth, 5 Nov. 2004; WAMC34900, 1 female, Exmouth Gulf, Western Australia, coll. S Morrison and P Unsworth, 12 Mar. 2004; WAM-C34938, 1 gravid female, Shark Bay, Western Australia, coll. S Morrison et al., 4 Oct. 2002; WAM-C5510, 1 male, Shark Bay, Western Australia, coll. S Morrison et al., 5 Mar. 2003; WAM-C44737, 1 female, Ningaloo Marine Park, Western Australia, coll. MP Salotti and SM Slack-Smith, 1 Feb. 2008.

SOUTH CHINA SEA (FAO Area 61): USNM1421161, 1 male, USNM1421181, 1 male, USNM1421182, 1 male, USNM1421185, 1 male, USNM1421187, 1 male, USNM1421191, 1 male, USNM1421194, 1 male, USNM1421195, 1 male, USNM1421186, 1 male, USNM121202, 1 male, USNM1421204, 1 male, USNM1421206, 1 male.

TAIWAN: UF29509, 1 female, Daxi Fishery Port; UF29511, 1 female, Daxi Fishery Port; UF29512, 1 female, Daxi Fishery Port; USNM1420827, 1 female, Daxi Fishery Port; USNM1420828, 1 male, Daxi Fishery Port; ZRC.1998.0186, 2 males, 1 female, Daxi Fishery Port, coll. PKL Ng, 3–4 Aug. 1996; ZRC.2016.0408, 4 males, 3 females, Daxi Fishery Port, coll. PKL Ng, 1 Jul. 2016.

##### Diagnosis.

Similar to *Monomiagladiator* except in the following morphological characters. Infraorbital margin granulate, terminating mesially in small triangular tooth, in line with rest of margin. Sixth pleomere (Fig. 2B, 3D–F) longer than wide, maximum width at base; anterior half of lateral margins convergent anteriorly, posterior half concave to straight, anterior and posterior halves separated by angular convexity. G1 (Fig. 3C, 4E–H) bent at 90° at midlength, tapering distally toward a minutely spatulate tip, slightly broader than immediate subdistal area. In fresh specimens, P5 propodus with dark purple spot on distal tip, distal one-third of P5 dactylus colored dark purple as well (Fig. 1E, F).

##### Remarks.

The primary morphological differences between *Monomiahaanii* (Stimpson, 1858) and *M.gladiator* (Fabricius, 1798) have already been discussed in the Remarks for the latter species. Yamaguchi and Baba (1993) had previously reported on the type material of *Amphitritehaanii* Stimpson, 1858, listing several syntypes collected by P.F. von Siebold from Japan in the years 1823 to 1829, deposited in the then Rijkmuseum van Natuurlijke Historie (RMNH) in Leiden, and preserved either dry or in alcohol. From these they selected a lectotype, a young male (42 by 24 mm; RMNH 379) preserved in alcohol. No further or detailed descriptions of the material were provided. Although they did provide photographs of several specimens, the lectotype was photographed still inside the bottle (viz. Yamaguchi and Baba, 1993: fig. 137A) and no definitive or diagnostic features of its morphology could be discerned. The collections of the RMNH are now housed in the Naturalis Biodiversity Center, Leiden, and photographs of the lectotype (Fig. 2) were kindly provided to the authors by Dr. Charles Fransen. From these photographs, the diagnostic morphology of the 6^th^ pleomere (Fig. 2B) and the 90-degree bend of the G1 (Fig. 2C) can already be observed even in such a young specimen. Furthermore, although the fresh coloration of this species was not recorded by either De Haan (1833) or Stimpson (1858), later observations of topotypic material show that the purple spots on the P5 propodus and dactylus, as well as the more profuse but scattered spotting on the dorsal carapace, and the dark colored spines on the anterior margin of the P1 merus are consistently observed (Fig. 1E, F; also Sakai, 1976: pl. 120 fig. 1; Miyake, 1983: pl. 29 fig. 1).

Stephenson and Cook (1973) had also previously synonymized *Portunuspseudoargentatus* Stephenson, 1961, under *P.haanii* on the basis of their similar morphology. We confirm that *P.pseudoargentatus* is a junior subjective synonym of *Monomiahaanii* (Stimpson, 1858) *sensu stricto* on the basis of similarities in the dentition of the infraorbital margin, the shape of the male 6^th^ pleomere (Fig. 3D), and the ~90° angle of the bend of the G1 at midlength (Fig. 4E; also Stephenson, 1961: fig. 2A). In a photograph of the presumably newly preserved holotype, the pigmented spots on the left P5 propodus and dactylus are still there (Stephenson, 1961: pl. 2 fig. 4), although these have since faded and can no longer be seen during the present examination of the holotype.

Specimens morphologically identifiable as *M.haanii* comprise a highly-supported clade that includes specimens from Japan, the type locality of *M.haanii*, as well as the holotype of *Portunuspseudoargentatus* Stephenson, 1961, and the Vietnamese specimens referred to by Chertoprud et al. (2012) (JX398094) and by Koch et al. (2017) and Koch and Duris (2018) (KY524463, KY524464) as "*Monomiapseudoargentata*". Both morphological and molecular phylogenetic analyses support the recognition of *Monomiahaanii* as a full species. Molecular results also support a morphological basis for synonymy of *P.pseudoargentatus* Stephenson, 1961, under *M.haanii*.

**Figure 5.**
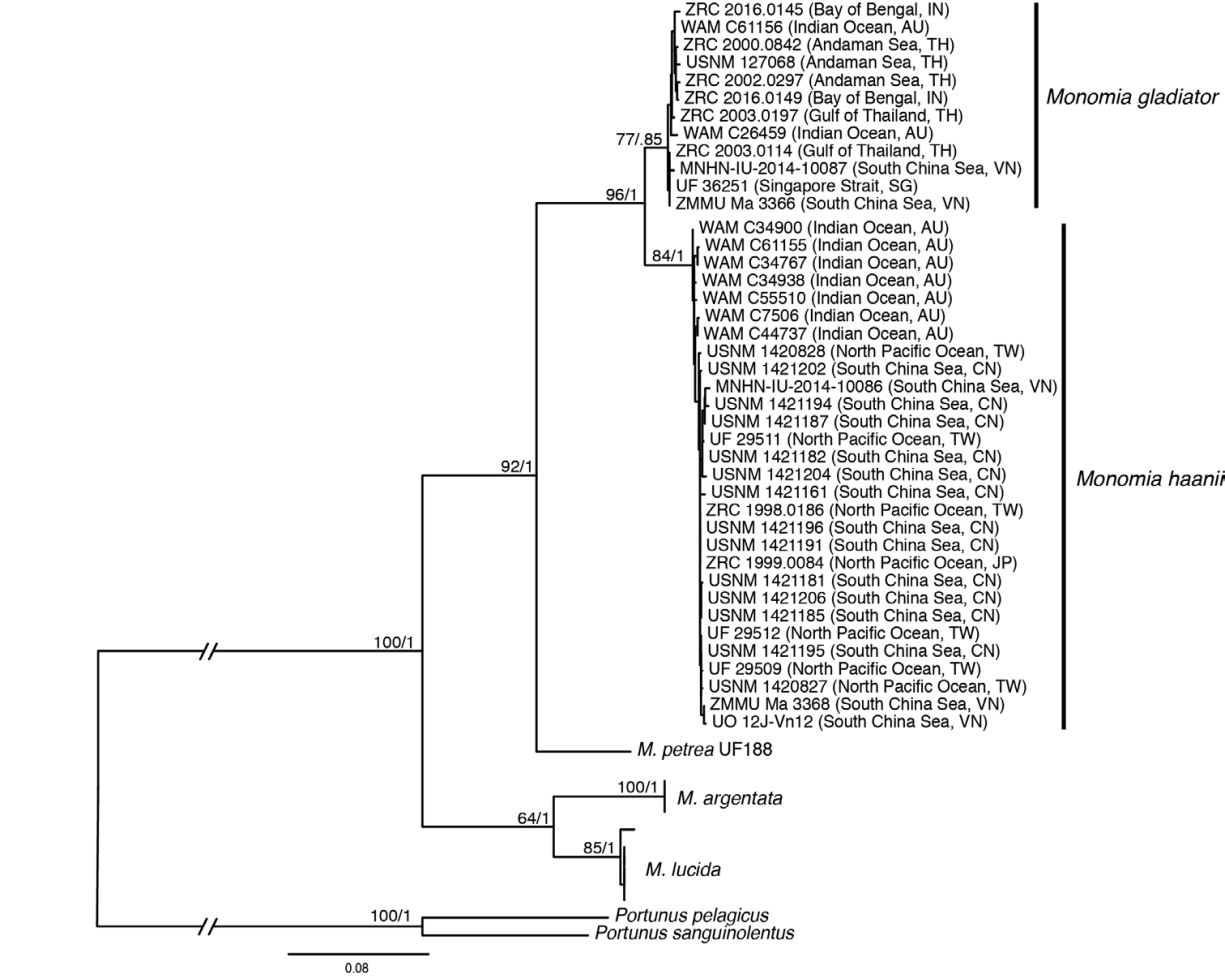
Maximum likelihood phylogram of three mitochondrial loci showing genetic distinction between *M.gladiator* and *M.haanii* along with three other members of the genus *Monomia*. Abbreviations: IOM = Institute of Oceanology and Museum, Nha Trang; MNHN = Muséum National d’Histoire Naturelle, Paris; NHM = The Natural History Museum, London; UF = University of Florida Natural History Museum, Gainesville; UO = University of Ostrava, Ostrava; USNM = United States National Museum, National Museum of Natural History, Washington, D.C.; WAM = Western Australian Museum, Perth; ZMMU = Zoological Museum of the Moscow University, Moscow; ZRC = Zoological Reference Collection, Lee Kong Chian Natural History Museum, Singapore; CN = China; IN = India; JP = Japan; SG = Singapore; TH = Thailand; TW = Taiwan; VN = Vietnam.

**Figure 6.**
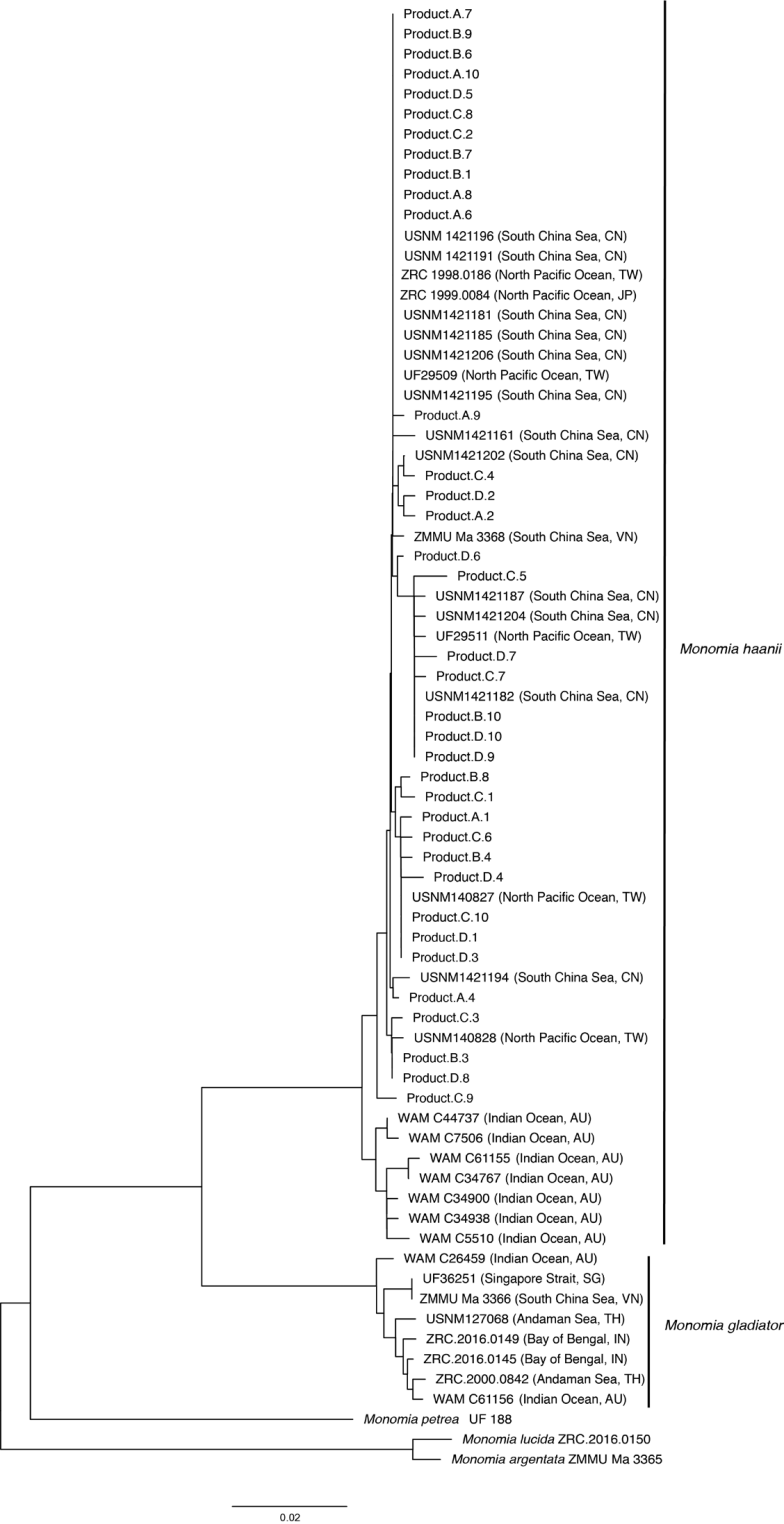
Neighbor joining phylogram of the barcode region of COI to visualize relationships between museum-vouchered reference specimens and 40 samples from four cans of pasteurized lump crabmeat (Product A–D) labeled “*Portunushaanii*” and/or “red swimming crab”

## Discussion

The need to address and resolve the *Portunusgladiator* species complex was brought about largely because of the incongruence in taxon names utilized by the scientific community and the seafood industry. This incongruence was highlighted by Warner et al. (2015) who matched sequences from crab cakes to a single sequence of “*P.pseudoargentatus*” and raised concerns that this was a species in the US food supply unknown to regulators. Subsequently, “*P.pseudoargentatus*” was added to The Seafood List (Food and Drug Administration 2015), based on that report. Pasteurized, lump crab meat labeled as “Portunushaanii” or “red swimming crab” is routinely imported into the United States; however, the species name *P.haanii* has been synonymised under *P.gladiator* for some time (Ng et al., 2008). As it turned out, this complex had a very complicated taxonomic past, but here we have used traditional morphological methods in concert with molecular phylogenetic analyses to establish the morphological and molecular boundaries between the two species we recognize herein, *Monomiagladiator* and *Monomiahaanii*.

Revising and describing the morphological differences between these two species was necessary to verify the identity of the individual specimens used to generate reference DNA sequences for identification of picked crab meat samples labelled as “*Portunushaanii*.” The morphological findings of two distinct species were corroborated in our multi-locus phylogenetic analysis that showed complete congruence between morphologically derived identity and genetic clade membership. This reciprocally informative approach has enabled us to confirm that the commercial products that we tested, that were labelled and sold as *Portunushaanii*, were in fact *Monomiahaanii* and should be labelled as such. Based on our findings, the FDA’s seafood labeling guidance to industry, The Seafood List, can be emended to reflect this current understanding of the species in question.

Commercially important species are often presumed to be well understood because they have tangible value, but in the case of decapod crustaceans molecular phylogenetic analyses are re-writing much of what carcinologists thought they knew about species-, genus-, and family-level relationships (Keenan et al. 1998; Ma et al. 2009; Lai et al. 2010; Bracken-Grissom et al. 2013; de Carvalho et al. 2013; Windsor and Felder 2014; Evans 2018; Tavares and Santana 2018). For example, Lai et al. (2010) determined that what was then known as *Portunuspelagicus* was actually a complex of four species; and the status of the genus *Penaeus* Fabricius, 1798, has been highly debated in the literature (Pérez Farfante and Kensley 1997; Lavery et al. 2004; Dall 2007; Flegel 2007; 2008; McLaughlin et al. 2008; Ma et al. 2009; Ma et al. 2011). These name changes are to be expected as new methods of DNA analysis are developed and applied to illuminate and clarify evolutionary relationships.

## Supplementary Material

XML Treatment for Monomia gladiator

XML Treatment for Monomia haanii

## Appendix 1

The types of *Monomiagladiator* (Fabricius, 1798) are currently deposited in the Natural History Museum of Denmark (NHMD) in Copenhagen. The lectotype (ZMUC-Cru 4705) has the corresponding catalog number, NHMD-82551. The paralectotypes (ZMUC-Cru 4704, 4706, and 4708) have the corresponding catalog numbers, NHMD-82550, -82552, and -82554, respectively (J. Olesen pers. comm.)

## References

[B1] Alcock A (1899) Materials for a carcinological fauna of India. No. 4. The Brachyura Cyclometopa. Part II. A revision of the Cyclometopa with an account of the families Portunidæ, Cancridæ and Corystidæ. Journal of the Asiatic Society of Bengal 68: 1–104.

[B2] Alibaba.com (2018) *Portunushaanii* crab https://www.alibaba.com/product-detail/Frozen-Portunus-Haanii-Crab_700629462.html?spm=a2700.9099375.35.7.fcd648d42A9aYD [accessed 6 December 2018]

[B3] Apakupakul K Siddall ME Burreson EM (1999) Higher level relationships of leeches (Annelida: Clitellata: Euhirudinea) based on morphology and gene sequences. Molecular Phylogenetics and Evolution 12, 350–359. 10.1006/mpev.1999.0639 10413628

[B4] Apel M Spiridonov VA (1998) Taxonomy and zoogeography of the portunid crabs (Crustacea: Decapoda: Brachyura: Portunidae) of the Arabian Gulf and adjacent waters. Fauna of Saudi Arabia 17: 159–331.

[B5] Balss H (1922) Ostasiatische Decapoden. IV. Die Brachyrhynchen (Cancridea). Archiv für Naturgeschichte 88(11): 94–166.

[B6] Barnard KH (1950) Descriptive catalogue of South African decapod Crustacea (crabs and shrimps). Annals of the South African Museum 38: 1–837.

[B7] Benedict JE (1893) Notice of the crustaceans collected by the U.S. Scientific Expedition to West Africa. Proceedings of the United States National Museum 16(949): 535–541.

[B8] Bhadra S (1998) Crustacea: Decapoda: Portunidae. In: Fauna of West Bengal, Part 10. Zoological Survey of India, State Fauna Series 3: 405–415.

[B9] Biju Kumar A Sushil Kumar M Raffi SM Ajmal Khan S (2007) Diversity of brachyuran crabs associated with trawl by-catch in Kerala coast, India. Indian Journal of Fisheries 54(3): 283–290.

[B10] Bracken-Grissom HD Cannon ME Cabezas P Feldmann RM Schweitzer CE Ahyong ST Felder DL Crandall KA (2013) A comprehensive and integrative reconstruction of evolutionary history for Anomura (Crustacea: Decapoda). BMC Evolutionary Biology 13: 128–156. 23786343 10.1186/1471-2148-13-128 PMC3708748

[B11] Carvalho FL de Pileggi LG Mantelatto FL (2013) Molecular data raise the possiblity of cryptic species in the Brazilian endemic prawn *Macrobrachiumpotiuna* (Decapoda, Palaemonidae). Latin American Journal of Aquatic Research 41: 707–717

[B12] Chertoprud ES Spiridonov VA Ponomarev SA Mokievsky VO (2012) Commercial crabs (CrustaceaDecapodaBrachyura) from Nhatrang Bay (Vietnam). In: Britayev TA Pavlov DS (Eds) Benthic fauna of the Bay of Nhatrang, southern Vietnam.KMK, Moscow, 296–344.

[B13] Colgan DJ McLauchlan A Wilson GDF Livingston SP Edgecombe GD Macaranas J Cassis G Gray MR (1998) Histone H3 and U2 snRNA DNA sequences and arthropod molecular evolution. Australian Journal of Zoology 46: 419–437.

[B14] Costa FO deWaard JR Boutillier J Ratnasingham S Dooh RT Hajibabaei M Hebert PDN (2007) Biological identifications through DNA barcodes: the case of the Crustacea. Canadian Journal of Fisheries and Aquatic Sciences 64: 272–295. 10.1139/f07-008

[B15] Crosnier A (1962) Crustacés Décapodes. Portunidae. Faune de Madagascar 16: 1–154.

[B16] Dai A Yang S (1991) Crabs of the China Seas. China Ocean Press: Beijing, 682 pp.

[B17] Dall W (2007) Recent molecular research on *Penaeus**sensu lato.* Journal of Crustacean Biology 27: 380–382. 10.1651/s-2803.1

[B18] Dev Roy MK Bhadra S (2005) Marine and estuarine crabs (Crustacea: Decapoda: Brachyura). In: Fauna of Andhra Pradesh (Part 5), Zoological Survey of India, State Fauna Series 5: 357–535.

[B19] Dev Roy MK Bhadra S (2011) Brachyuran crabs (Crustacea: Decapoda: Brachyura). In: Fauna of Tamil Nadu, Zoological Survey of India, State Fauna Series 17(2): 109–269.

[B20] Eischeid AC Stadig SR Handy SM Fry FS Deeds J (2016) Optimization and evaluation of a method for the generation of DNA barcodes for the identification of crustaceans. Lwt-Food Science and Technology 73: 357–367. 10.1016/j.lwt.2016.06.033

[B21] Evans N (2018) Molecular phylogenetics of swimming crabs (Portunoidea Rafinesque, 1815) supports a revised family-level classification and suggests a single derived origin of symbiotic taxa. PeerJ 6: e4260. 10.7717/peerj.4260 PMC5786103 29379685

[B22] Fabricius JC (1793) Entomologica Systematica Emendata et Aucta. Secundum. Classes, Ordines, Genera, Species. Adjectis. Synonimis, Locis, Observationibus, Descriptionibus. Tom. II. Hafniae Impensis Christ. Gottl. Proft. I–VIII, 1–519. 10.5962/bhl.title.125869

[B23] Fabricius JC (1798) Supplementum Entomologiae Systematicae. Proft et Storch, Hafniae, 572 pp.

[B24] Fishsource (2016) Warty Swimming Crab: China. https://www.fishsource.org/fishery_page/3725 [accessed 6 December 2018]

[B25] Flegel TW (2007) The right to refuse revision in the genus *Penaeus*. Aquaculture 264: 2–8. 10.1016/j.aquaculture.2006.12.013

[B26] Flegel TW (2008) Confirmation of the right to refuse revision in the genus *Penaeus*. Aquaculture 280: 1–4. 10.1016/j.aquaculture.2008.04.029

[B27] Food and Agriculture Organization of the United Nations (2010-2018) FAO FishFinder: Aquatic Species Fact Sheets. http://www.fao.org/fishery/species/search/en [accessed 17 November 2018]

[B28] Food and Drug Administration (2012) The Seafood List: FDA Guide to Acceptable Market Names for Food Fish Sold in Interstate Commerce. http://www.fda.gov/Food/GuidanceComplianceRegulatoryInformation/GuidanceDocuments/Seafood/ucm113260 [accessed 08 November 2018]

[B29] Food and Drug Administration (2015) The FDA Seafood List: Updates for 2015. https://wayback.archive-it.org/7993/20170404003109/https://www.fda.gov/Food/GuidanceRegulation/GuidanceDocumentsRegulatoryInformation/Seafood/ucm453865.htm [accessed 10 December 2018]

[B30] Food and Drug Administration (2017) Reference Standard Sequence Library (RSSL) for Seafood Identification. https://www.accessdata.fda.gov/scripts/fdcc/?set=seafood_barcode_data [accessed 5 July 2018]

[B31] Fourmanoir P (1954) Crabes de la côte ouest de Madagascar. Le Naturaliste Malgache 6: 2–16.

[B32] Geller J Meyer C Parker M Hawk H (2013) Redesign of PCR primers for mitochondrial cytochrome c oxidase subunit I for marine invertebrates and application in all-taxa biotic surveys. Molecular Ecology Resources 13: 851–861. 10.1111/1755-0998.12138 23848937

[B33] Gistel J (1848) Naturgeschichte des Thierreiches für höhere Schulen bearbeitet. Stuttgart, xvi, 216 pp., pls. 211–232.

[B34] Haan W de (1833–1850) Crustacea. In: Siebold PFv (Ed.) Fauna Japonica sive Descriptio Animalium, Quae in Itinere per Japoniam, Jussu et Auspiciis Superiorum, qui Summum in India Batava Imperium Tenent, Suscepto, Annis 1823–1830 Collegit, Noitis, Observationibus et Adumbrationibus Illustravit.Lugduni-Batavorum, Leiden, i–xvii, i–xxxi, ix–xvi, 1–243.

[B35] Handy SM Deeds JR Ivanova NV Herbert PDN Hanner RH Ormos A Weigt LA Moore MM Yancy HF (2011) A single-laboratory validated method for the generation of DNA barcodes for the identification of fish for regulatory compliance. Journal of AOAC International 94: 201–210. 21391497

[B36] Henderson JR (1893) A contribution to Indian carcinology. Transactions of the Linnean Society of London, ser. 2, Zoology 5(10): 325–458, pls 36–40. 10.1111/j.1096-3642.1893.tb00653.x

[B37] Herbst JFW (1782–1804) Versuch einer Naturgeschichte der Krabben und Krebse nebst einer systematischen Beschreibung ihrer verschieden Arten. Berlin und Stralsund, 3 vols, 72 pls. 10.5962/bhl.title.62813

[B38] Huang J-F Yu H-P (1997) Illustrations of swimming crabs from Taiwan. National Museum of Marine Biology & Aquarium, 181 pp. (in Chinese).

[B39] ICZN [International Commission for Zoological Nomenclature] (1999) International Code of Zoological Nomenclature, 4^th^ ed. International Trust for Zoological Nomenclature, London.

[B40] Jeyabaskaran R Ajmal Khan S Ramaiyan V. (2000) Brachyuran crabs of Gulf of Mannar. Biodiversity Project on Gulf of Mannar Biosphere Reserve. Parangipettai: Annamallai University, 99 pp. [78 pls]

[B41] Katoh K Standley DM (2013) MAFFT multiple sequence alignment software version 7: improvements in performance and usability. Molecular Biology and Evolution 30: 772–780. 10.1093/molbev/mst010 23329690 PMC3603318

[B42] Keenan CP Davie PJF Mann DL (1998) A revision of the genus *Scylla* de Haan, 1833 (Crustacea: Decapoda: Brachyura: Portunidae). Raffles Bulletin of Zoology 46: 217–245.

[B43] Kim HS Chang CY (1985) The brachyuran crabs of Cheju Island, Korea (Crustacea: Decapoda). Korean Journal of Systematic Zoology 1(1–2): 41–60.

[B44] Ko H-S Lee S-H (2013) Arthropoda: Malacostraca: Decapoda: Brachyura: Cancridae, Cheiragonidae, Dorippidae, Euryplacidae, Goneplacidae, Hymenosomatidae, Portunidae. Crabs and zoeas III. Invertebrate Fauna of Korea 21(30): 1–76. [8 pls]

[B45] Koch M Ďuriš Z (2018) *Monomialucida* sp. nov., a new swimming crab (Crustacea: Decapoda: Portunidae) from the South China Sea. Zootaxa 4387: 567–579. 10.11646/zootaxa.4387.3.9 29690481

[B46] Koch M Kamanli SA Crimmen O Lin C–W Clark PF Ďuriš Z (2017) The identity of *Monomiaargentata* (Crustacea : Brachyura : Portunidae) resolved by X-ray, computed tomography scanning and molecular comparisons. Invertebrate Systematics 31: 797–811. 10.1071/IS16058

[B47] Krishnamoorthy P (2009) Brachyuran crabs from the collections of the Marine Biological Centre. Records of the Zoological Survey of India, Occasional Paper 304: 1–46.

[B48] Kumar S Stecher G Tamura K (2016) MEGA 7: Molecular Evolutionary Genetics Analysis version 7.0. Molecular Biology and Evolution 33: 1870–1874. 10.1093/molbev/msw054 27004904 PMC8210823

[B49] Lai JCY Ng PKL Davie P (2010) A revision of the *Portunuspelagicus* (Linnaeus, 1758) species complex (Crustacea: Brachyura: Portunidae), with the recognition of four species. Raffles Bulletin of Zoology 58: 199–237.

[B50] Lanchester WF (1902) On the Crustacea collected during the “Skeat” Exp[edition to the Malay Peninsula, together with a note on the genus *Actaeopsis*. Part I. Brachyura, Stomatopoda, and Macrura. Proceedings of the Zoological Society of London 1901(2): 534–574. [pls 33, 34] 10.1111/j.1469-7998.1902.tb08187.x

[B51] Lanfear R Calcott B Ho SY Guindon S (2012) Partitionfinder: combined selection of partitioning schemes and substitution models for phylogenetic analyses. Molecular Biology and Evolution 29: 1695–1701. 10.1093/molbev/mss020 22319168

[B52] Lanfear R Frandsen PB Wright AM Senfeld T Calcott B (2016) PartitionFinder 2: New Methods for selecting partitioned models of evolution for molecular and morphological phylogenetic analyses. Molecular Biolology and Evololution 34: 772–773. 10.1093/molbev/msw260 28013191

[B53] Latreille PA (1825) Histoire Naturelle. Entomologie, ou Histoire naturelle des Crustacés, des Arachnides et des Insectes. Agasse Imprimeur-Libraire, Paris, 832 pp.

[B54] Laurie RD (1906) Report on the Brachyura collected by Professor Herdman, at Ceylon, in 1902. In: Herdman W. A. (ed.) Report to the Government of Ceylon on the Pearl Oyster Fisheries of the Gulf of Manaar with Supplementary Reports Upon the Marine Biology of Ceylon by Other Naturalists, Part 5, Suppl. Rep.40: 349–432. [pls 1, 2]

[B55] Lavery S Chan T-Y Tam YK Chu KH (2004) Phylogenetic relationships and evolutionary history of the shrimp genus *Penaeus* s.l. derived from mitochondrial DNA. Molecular Phylogenetics and Evolution 31: 39–49. 10.1016/j.ympev.2003.07.015 15019607

[B56] Lin CC (1949) A catalogue of brachyurous Crustacea of Taiwan. Quarterly Journal of the Taiwan Museum 2(1): 10–33.

[B57] Ma KY Chan T-Y Chu KH (2011) Refuting the six-genus classification of *Penaeus* s.l. (Dendrobranchiata, Penaeidae): a combined analysis of mitochondrial and nuclear genes. Zoologica Scripta 40: 498–508. 10.1111/j.1463-6409.2011.00483.x

[B58] Ma KY Chan T-Y Chu KH (2009) Phylogeny of penaeoid shrimps (Decapoda: Penaeoidea) inferred from nuclear protein-coding genes. Molecular Phylogenetics and Evolution 53: 45–55. 10.1016/j.ympev.2009.05.019 19477284

[B59] Man JG De (1888) Report on the Podophthalmous Crustacea of the Mergui Archipelago, collected for the trustees of the Indian Museum, Calcutta, by Dr. John Anderson, F.R.S., Superintendent of the Museum.—Part IV. Journal of the Linnean Society of London (Zoology), 22(139): 177–240. [pls 13–15] 10.1111/j.1096-3642.1888.tb00031.x

[B60] Mantelatto FL Robles R (2007) Molecular phylogeny of the western Atlantic species of the genus *Portunus* (Crustacea, Brachyura, Portunidae). Zoological Journal of the Linnean Society 150: 211–220. 10.1111/j.1096-3642.2007.00298.x

[B61] Mantelatto FL Robles R Schubart CD Felder DL (2009) Molecular phylogeny of the genus *Cronius* Stimpson, 1860, with reassignment of *C.tumidulus* and several American species of *Portunus* to the genus *Achelous* De Haan, 1833 (Brachyura: Portunidae). Decapod Crustacean Phylogenetics. CRC Press, 567–579. 10.1201/9781420092592-c29

[B62] McLaughlin PA Lemaitre R Ferrari FD Felder DL Bauer RT (2008) A reply to T.W. Flegel. Aquaculture 275: 370–373. 10.1016/j.aquaculture.2007.12.020

[B63] Medlin LK Elwood HJ Stickles S and Sogin (1988) The characterization of enzymatically amplified eukaryotic 16S-like rRNA-coding regions. Gene 71: 491–499. 10.1016/0378-1119(88)90066-2 3224833

[B64] Miers EJ (1886) Report on the Brachyura collected by H.M.S. *Challenger* during the years 1873–1876. In: Murray J (Ed.) Zoology. Neill and Company, Edinburgh, 362 pp.

[B65] Milne-Edwards A (1861) Etudes zoologiques sur les crustacés récents de la famille des portuniens. Nouvelles archives du Muséum d’Histoire naturelle, Paris 10: 309–421. [pls 28–38] 10.5962/bhl.title.10629

[B66] Milne-Edwards H (1834) Histoire naturelle des Crustacés, comprenant l’anatomie, la physiologie et la classification de ces animaux. Roret, Paris, (1)468, (462)532, (463)638 pp. 10.5962/bhl.title.39738

[B67] Miyake S (1961) A list of the decapod Crustacea of the Sea of Ariaké, Kyushu. Records of Oceanographic Works in Japan 5: 165–178.

[B68] Miyake S (1983) Japanese crustacean decapods and stomatopods in color. Vol. II. Brachyura (crabs), i–viii, 1–277, pls. 1–64. Hoikusha, Osaka. [in Japanese]

[B69] Mokady O Rozenblatt S Graur D Loya Y (1994) Coral-host specificity of red sea *Lithophaga* bivalves: interspecific and intraspecific variation in 12S mitochondrial ribosomal RNA. Molecular Marine Biology and Biotechnology 3: 158–164. 7522832

[B70] Monterey Bay Aquarium Seafood Watch (2013) Blue and red swimming crab *Portunuspelagicus* and *Portunushaanii*. Indonesia, India, China, Viet Nam, Thailand. http://www.seafoodwatch.org/-/m/sfw/pdf/reports/c/mba_seafoodwatch_swimmercrab_report.pdf [accessed 05 December 2018]

[B71] Moosa MK (1996) CrustaceaDecapoda: Deep-water swimming crabs from the South-West Pacific, particularly New Caledonia (Brachyura, Portunidae). In: Crosnier A (Ed.) Résultats des Campagnes MUSORSTOM, vol 15.Mémoires du Muséum national d’Histoire naturelle 168: 503–530.

[B72] Muller F (1887) Zur Crustaceenfauna von Trincomali. Verhandlungen der Naturforschenden Gesellschaft in Basel 8(2): 470–485. [pls IV, V] 10.5962/bhl.part.19447

[B73] Müller OF (1771) Von Würmern des süssen und salzigen Wassers. Copenhagen, 200 pp. 10.5962/bhl.title.14428

[B74] Ng PKL Guinot D Davie PJF (2008) Systema Brachyourum: Part I. An annotated checklist of extant brachyuran crabs of the world. Raffles Bulletin of Zoology Supplement 17: 1–286.

[B75] Ng PKL Shih H-T Ho P-H Wang C-H (2017) An updated annotated checklist of brachyuran crabs from Taiwan (Crustacea: Decapoda). Journal of the National Taiwan Museum 70(3 & 4): 1–185.

[B76] Ng PKL Wang C-H Ho P-H Shih H-T (2001) An annotated checklist of brachyuran crabs from Taiwan (Crustacea: Decapoda). National Taiwan Special Publication Series, No. 11. National Taiwan Museum, Taipei, 86 pp.

[B77] Ortmann A (1893) Die Decapoden-Krebse des Strassburger Museums, mit besonderer Beriicksichtigung der von Herrn Dr. Döderlein bei Japan und bei Riu-Kiu Inseln gesammelten und zur Zeit im Strassburger Museum aufbewahrten Formen. VII. Brachyura. II. Cyclometopa. Zoologische Jahrbücher für Systematik 7: 411–495. [pl. 17] 10.5962/bhl.part.24064

[B78] Palumbi SR (1996) Nucleic acids II: the polymerase chain reaction. In: Hillis DM, Moritz C, Mable BK (Eds) Sinauer, Sunderland, 205-247.

[B79] Parisi B (1916) I decapodi giapponesi del Museo di Milano. IV. Cyclometopa. Atti della Società Italiana di Scienze naturali 55: 5–42. [pls 7–11]

[B80] Pérez Farfante I Kensley B (1997) Penaeoid and sergestoid shrimps and prawns of the world. Keys and diagnoses for the families and genera. Mémoires du Muséum national d’Histoire naturelle 175: 1–233.

[B81] Rathbun MJ (1896) The genus *Callinectes*. Proceedings of the United States National Museum 18: 349–375. 10.5479/si.00963801.18-1070.349

[B82] Rathbun MJ (1902) Japanese stalk-eyed crustaceans. Proceedings of the United States National Museum 26(1307): 23–55. 10.5479/si.00963801.26-1307.23

[B83] Rathbun MJ (1910) The Brachyura. The Danish Expedition to Siam, 1899–1900. V. Kongelige Danske Videnskabernes Selskabs Skrifter, Kjøbenhavn (7) 5(4): 301–367. [pls 1, 2]

[B84] Richters F (1880) Decapoda. In: Möbius K (Ed.) Beiträge zur Meeresfauna der Insel Mauritius und der Seychellen bearbeitet von K. Möbius, F. Richters und E. von Martens nach Sammlungen, angelegt auf einer Reise nach Mauritius von K. Möbius. Berlin: Verlag der Gutmann’schen Buchhandlung.139–178. [pls 15–18]

[B85] Ronquist F Huelsenbeck JP (2003) MrBayes 3: Bayesian phylogenetic inference under mixed models. Bioinformatics 19: 1572–1574. 10.1093/bioinformatics/btg180 12912839

[B86] Sakai T (1934) Brachyura from the coast of Kyushu, Japan. Scientific Reports of the Tokyo Bunrika Daigaku, sec. B 1: 281–330.

[B87] Sakai T (1936) Crabs of Japan. Tokyo, 1–239, 1–12, pls 1–66, frontispiece. [in Japanese]

[B88] Sakai T (1939) Studies on the Crabs of Japan IV. Brachygnatha, Brachyrhyncha. Vol. 3. Yokendo Co., Ltd, Tokyo, 365–741.

[B89] Sakai T (1965) The Crabs of Sagami Bay: Collected by His Majesty the Emperor of Japan. Maruzen Co, Ltd, Tokyo, i–xvi, 1–206, 1–92, 1–32, pls 1–100.

[B90] Sakai T (1976) Crabs of Japan and the Adjacent Seas. Kodansha Ltd, Tokyo, 773 pp.

[B91] Schubart CD Reuschel S (2009) A proposal for a new classification of Portunoidea and Cancroidea (Brachyura: Heterotremata) based on two independent molecular phylogenies. In: Martin JW Crandall KA Felder DL (Eds) Decapod Crustacean Phylogenetics.CRC Press, Taylor and Francis Group, Boca Raton, London, New York, 533–549. 10.1201/9781420092592-c27

[B92] Shull HC Perez-Losada M Blair D Sewell K Sinclair EA Lawler S Ponniah M Crandall KA (2005) Phylogeny and biogeography of the freshwater crayfish *Euastacus* (Decapoda: Parastacidae) based on nuclear and mitochondrial DNA. Molecular Phylogenetics and Evolution 37: 249–263. 10.1016/j.ympev.2005.04.034 16029952

[B93] Spiridonov VA Neretina TV Schepetov D (2014) Morphological characterization and molecular phylogeny of Portunoidea Rafinesque, 1815 (CrustaceaBrachyura): Implications for understanding evolution of swimming capacity and revision of the family-level classification. Zoologischer Anzeiger 253: 404–429. 10.1016/j.jcz.2014.03.003

[B94] Stamatakis A (2006) RAxML-VI-HPC: maximum likelihood-based phylogenetic analyses with thousands of taxa and mixed models. Bioinformatics 22: 2688–2690. 10.1093/bioinformatics/btl446 16928733

[B95] Stebbing TRR (1915) South African Crustacea (Part VIII of S. A. Crustacea, for the marine investigations in South Africa). Annals of the South African Museum 15(2): 57–104. [pls 13-25] 10.5962/bhl.part.22195

[B96] Stephenson W (1961) The Australian portunids (Crustacea: Portunidae) V. Recent collections. Australian Journal of Marine and Freshwater Research 12: 98–128. 10.1071/MF9610092

[B97] Stephenson W (1972a) Portunid crabs from the Indo-West-Pacific and Western America in the Zoological Museum, Copenhagen (Decapoda, Brachyura, Portunidae). Steenstrupia 2(9): 127–156.

[B98] Stephenson W (1972b) An annotated check list and key to the Indo-West Pacific swimming crabs (Crustacea: Decapoda: Portunidae). Bulletin of the Royal Society of New Zealand 10: 1–62.

[B99] Stephenson W (1975) Biological results of the Snellius Expedition, XXVI. The Portunidae (Decapoda - Brachyura) of the Snellius Expedition (Part II). Zoologische Mededelingen 49(14): 173–206.

[B100] Stephenson W Campbell BM (1959) The Australian portunids (Crustacea: Portunidae). Australian Journal of Marine and Freshwater Research 10: 84–124. 10.1071/MF9590084

[B101] Stephenson W Cook S (1973) Studies of *Portunusgladiator* complex and related species of *Portunus* (Crustacea: Decapoda). Memoirs of the Queensland Museum 16: 415–434.

[B102] Stephenson W Rees M (1967) Portunid crabs from the International Indian Ocean Expedition in the Smithsonian Collections (Crustacea: Portunidae). Proceedings of the United States National Museum 122(3599): 1–33. 10.5479/si.00963801.122-3599.1

[B103] Stephenson W Rees M (1967b) Some portunid crabs from the Pacific and Indian Oceans in the collections of the Smithsonian Institution. Proceedings of the United States National Museum 120: 1–114. 10.5479/si.00963801.120-3556.1

[B104] Stephenson W Rees M (1968) The *Endeavour* and other Australian Museum collections of portunid crabs (Crustacea, Decapoda, Portunidae). Records of the Australian Museum 27(13): 285–298. 10.3853/j.0067-1975.27.1968.447

[B105] Stimpson W (1858) Prodromus descriptionis animalium evertebratorum, quae in Expeditione ad Oceanum Pacificum Septentrionalem, a Republica Federata missa, Cadwaladaro Ringgold et Johanne Rodgers Ducibus, observavit et descripsit. Pars IV. CrustaceaCancroidea et Corystoidea. Proceedings of the Academy of Natural Sciences of Philadelphia 10: 31–40. 10.5962/bhl.title.51447

[B106] Stimpson W (1907) Report on the Crustacea (Brachyura and Anomura) Collected by the North Pacific Exploring Expedition, 1853–1856. Smithsonian Miscellaneous Collections 49: 1–240. 10.5962/bhl.title.51448

[B107] Takeda M (1989) Shallow-water crabs from Oshima Passage between Amami-Oshima and Kakero-jima Islands, the Northern Ryukyu Islands. Memoirs of the National Science Museum, Tokyo 22: 135–184.

[B108] Takeda M Miyake S (1968) Crabs from the East China Sea, I. Corystoidea and Brachygnatha Brachyrhyncha. Journal of the Faculty of Agriculture, Kyushu University 14(4): 541–582.

[B109] Tavares M Santana W (2018) Refining the genus *Rochinia* A. Milne-Edwards, 1875: reinstatement of *Scyramathia* A. Milne-Edwards, 1880 and *Anamathia* Smith, 1885, and a new genus for *Amathiacrassa* A. Milne-Edwards, 1879, with notes on its ontogeny (Crustacea: Brachyura: Epialtidae). Zootaxa 4418: 201–227. 10.11646/zootaxa.4418.3.1 30313582

[B110] Trivedi JN Trivedi DJ Vachhrajani KD Ng PKL (2018) An annotated checklist of the marine brachyuran crabs (Crustacea: Decapoda: Brachyura) of India. Zootaxa 4502(1): 1–83. 10.11646/zootaxa.4502.1.1 30486044

[B111] Vaidya G Lohman DJ Meier R (2011) SequenceMatrix: concatenation software for the fast assembly of multi-gene datasets with character set and codon information. Cladistics 27: 171–180. 10.1111/j.1096-0031.2010.00329.x 34875773

[B112] Warner K Lowell B Disla C Ortenzi K Savits J Hirshfield M (2015) Oceana reveals mislabeling of iconic Chesapeake blue crab. Oceana, 1–15. https://oceana.org/sites/default/files/crab_testing_report_final_3.27.15.pdf

[B113] Wee DPC Ng PKL (1995) Swimming crabs of the genera *Charybdis* De Haan, 1833, and *Thalamita* Latreille, 1829 (Crustacea: Decapoda: Brachyura: Portunidae) from Peninsular Malaysia and Singapore. Raffles Bulletin of Zoology, Suppl. 1: 1–128.

[B114] Windsor AM Felder DL (2014) Molecular phylogenetics and taxonomic reanalysis of the family Mithracidae MacLeay (Decapoda: Brachyura: Majoidea). Invertebrate Systematics 28: 145–173. 10.1071/is13011

[B115] Yamaguchi T Baba K (1993) Crustacean specimens collected in Japan by Ph. F. von Siebold an H. Bürger and held by the National Natuurhistorisch Museum in Leiden and other museums. In: Yamaguchi T (Ed.) Ph F von Siebold and Natural History of Japan, Crustacea.The Carcinological Society of Japan, Tokyo, 145–570.

[B116] Yang S Dai A (1994) Further notes on the crabs (BrachyuraCrustacea) from Nansha Islands and its adjacent waters. Marine Fauna and Flora and Biogeography of the Nansha Islands and Neighbouring Waters, 125–148. [in Chinese]

